# Microcanonical and resource-theoretic derivations of the thermal state of a quantum system with noncommuting charges

**DOI:** 10.1038/ncomms12051

**Published:** 2016-07-07

**Authors:** Nicole Yunger Halpern, Philippe Faist, Jonathan Oppenheim, Andreas Winter

**Affiliations:** 1 Institute for Quantum Information and Matter, Division of Physics, Mathematics, and Astronomy, California Institute of Technology, Pasadena, California 91125, USA; 2 Institute for Theoretical Physics, Department of Physics, ETH Zürich, 8093 Zürich, Switzerland; 3 Department of Physics and Astronomy, University College London, Gower Street, London WC1E 6BT, UK; 4 Departament de Física, Grup d'Informació Quántica, Universitat Autònoma de Barcelona, ES-08193 Barcelona, Spain; 5 ICREA, Pg. Lluís Companys 23, 08010 Barcelona, Spain

## Abstract

The grand canonical ensemble lies at the core of quantum and classical statistical mechanics. A small system thermalizes to this ensemble while exchanging heat and particles with a bath. A quantum system may exchange quantities represented by operators that fail to commute. Whether such a system thermalizes and what form the thermal state has are questions about truly quantum thermodynamics. Here we investigate this thermal state from three perspectives. First, we introduce an approximate microcanonical ensemble. If this ensemble characterizes the system-and-bath composite, tracing out the bath yields the system's thermal state. This state is expected to be the equilibrium point, we argue, of typical dynamics. Finally, we define a resource-theory model for thermodynamic exchanges of noncommuting observables. Complete passivity—the inability to extract work from equilibrium states—implies the thermal state's form, too. Our work opens new avenues into equilibrium in the presence of quantum noncommutation.

## Abstract (web summary)

A central concept in thermodynamics is the thermal state, which is the one towards which the system relaxes. Here, the authors derive the same state, through three different approaches, in the case of a quantum system whose conserved quantities correspond to operators that do not commute with one another.

## Graphical abstract

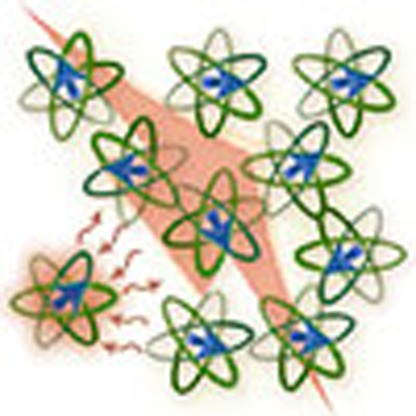

Recently reignited interest in quantum thermodynamics has prompted information-theoretic approaches to fundamental questions^1^,^2^,^3^,^4^. The role of entanglement, for example, has been clarified with canonical typicality^5^,^6^,^7^,^8^. Equilibrium-like behaviours have been predicted^9^,^10^,^11^,^12^ and experimentally observed in integrable quantum gases^13^,^14^.

Thermodynamic resource theories offer a powerful tool for analysing fundamental properties of the thermodynamics of quantum systems. Heat exchanges with a bath are modelled with ‘free states' and ‘free operations'^15^,^16^,^17^,^18^. These resource theories have been extended to model exchanges of additional physical quantities, such as particles and angular momentum^18^,^19^,^20^,^21^,^22^.

A central concept in thermodynamics and statistical mechanics is the thermal state. The thermal state has several important properties. First, typical dynamics evolve the system towards the thermal state. The thermal state is the equilibrium state. Second, consider casting statistical mechanics as an inference problem. The thermal state is the state that maximizes the entropy under constraints on physical quantities^23^,^24^. Third, consider the system as interacting with a large bath. The system-and-bath composite occupies a microcanonical state. Physical observables of the composite, such as the total energy and total particle number, have sharply defined values. The system's reduced state is the thermal state. Finally, in a resource theory, the thermal state is the only completely passive state. No work can be extracted from any number of copies of the thermal state^25^,^26^.

If a small system exchanges heat and particles with a large environment, the system's thermal state is a grand canonical ensemble: *e*^−*β*(*H*−*μN*)^/*Z*. The system's Hamiltonian and particle number are represented by *H* and *N*. *β* and *μ* denote the environment's inverse temperature and chemical potential. The partition function *Z* normalizes the state. The system-and-bath dynamics conserves the total energy and total particle number. More generally, subsystems exchange conserved quantities, or ‘charges,' *Q*_*j*_, *j*=1, 2,…*c*. To these charges correspond generalized chemical potentials *μ*_*j*_. The *μ*_*j*_'s characterize the bath.

We address the following question. Suppose that the charges fail to commute with each other: [*Q*_*j*_, *Q*_*k*_]≠0. What form does the thermal state have? We call this state ‘the non-Abelian thermal state' (NATS). Jaynes applied the principle of maximum entropy to this question^24^. He associated fixed values *v*_*j*_ with the charges' expectation values. He calculated the state that, on satisfying these constraints, maximizes an entropy. This thermal state has a generalized Gibbs form:


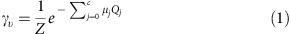


wherein the *v*_*j*_'s determine the *μ*_*j*_'s.

Our contribution is a mathematical, physically justified derivation of the thermal state's form for systems whose dynamics conserve noncommuting observables. We recover the state (1) via several approaches, demonstrating its physical importance. We address puzzles raised in refs 21, 27 about how to formulate a resource theory in which thermodynamic charges fail to commute. Closely related, independent work was performed by Guryanova *et* al.^28^ We focus primarily on the nature of passive states. Guryanova *et al*., meanwhile, focus more on the resource theory for multiple charges and on trade-offs amongst types of charge extractions.

In this paper, we derive the NATS's form from a micro-canonical argument. A simultaneous eigenspace of all the noncommuting physical charges might not exist. Hence, we introduce the notion of an approximate microcanonical subspace. This subspace consists of the states in which the charges have sharply defined values. We derive conditions under which this subspace exists. We show that a small subsystem's reduced state lies, on average, close to *γ*_**v**_. Second, we invoke canonical typicality^7^,^8^. If the system-and-bath composite occupies a random state in the approximate microcanonical subspace, we argue, a small subsystem's state likely lies close to the NATS. Typical dynamics are therefore expected to evolve a well-behaved system's state towards the NATS. Third, we define a resource theory for thermodynamic exchanges of noncommuting conserved charges. We extend existing resource theories to model the exchange of noncommuting quantities. We show that the NATS is the only possible free state that renders the theory nontrivial: Work cannot be extracted from any number of copies of *γ*_**v**_. We show also that the NATS is the only state preserved by free operations. From this preservation, we derive ‘second laws' that govern state transformations. This work provides a well-rounded, and novelly physical, perspective on equilibrium in the presence of quantum noncommutation. This perspective opens truly quantum avenues in thermodynamics.

## Results

### Overview

We derive the NATS's form via three routes: from a microcanonical argument; from a dynamical argument built on canonical typicality; and from complete passivity in a resource theory. Details appear in Supplementary Notes 1–3.

### Microcanonical derivation

In statistical mechanics, the form *e*^−*β*(*H*−*μN*)^/*Z* of the grand canonical ensemble is well known to be derivable as follows. The system of interest is assumed to be part of a larger system. Observables of the composite have fixed values *v*_*j*_. For example, the energy equals *E*_0_, and the particle number equals *N*_0_. The microcanonical ensemble is the whole-system state spread uniformly across these observables' simultaneous eigenspace. Tracing out the environmental degrees of freedom yields the state *e*^−*β*(*H*−*μN*)^/*Z*.

**Figure 1:**
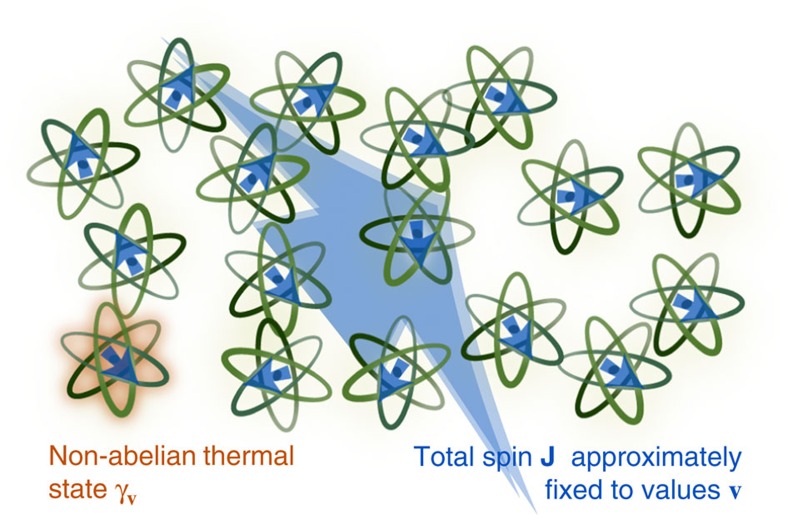
Non-Abelian thermal state. We derive the form of the thermal state of a system that has charges that might not commute with each other. Example charges include the components *J*_*i*_ of the spin **J**. We derive the thermal state's form by introducing an approximate microcanonical state. An ordinary microcanonical ensemble could lead to the thermal state's form if the charges commuted: suppose, for example, that the charges were a Hamiltonian *H* and a particle number *N* that satisfied [*H*, *N*]=0. Consider many copies of the system. The composite system could have a well-defined energy *E*_tot_ and particle number *N*_tot_ simultaneously. *E*_tot_ and *N*_tot_ would correspond to some eigensubspace 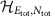 shared by the total Hamiltonian and the total-particle-number operator. The (normalized) projector onto 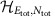 would represent the composite system's microcanonical state. Tracing out the bath would yield the system's thermal state. But the charges *J*_*i*_ under consideration might not commute. The charges might share no eigensubspace. Quantum noncommutation demands a modification of the ordinary microcanonical argument. We define an approximate microcanonical subspace 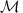. Each state in 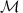 simultaneously has almost-well-defined values of noncommuting whole-system charges: measuring any such whole-system charge has a high probability of outputting a value close to an ‘expected' value analogous to *E*_tot_ and *N*_tot_. We derive conditions under which the approximate microcanonical subspace 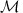 exists. The (normalized) projector onto 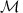 represents the whole-system state. Tracing out most of the composite system yields the reduced state of the system of interest. We show that the reduced state is, on average, close to the NATS. This microcanonical derivation of the NATS's form strengthens the link between Jaynes's information-theoretic derivation and physics.

We derive the NATS's form similarly. Crucially, however, we adapt the above strategy to allow for noncommuting observables. Observables might not have well-defined values *v*_*j*_ simultaneously. Hence a microcanonical ensemble as discussed above, suitable for commuting observables, may not exist. We overcome this obstacle by introducing an approximate microcanonical ensemble Ω. We show that, for every state satisfying the conditions of an approximate microcanonical ensemble, tracing out most of the larger system yields, on average, a state close to the NATS. We exhibit conditions under which an approximate microcanonical ensemble exists. The conditions can be satisfied when the larger system consists of many noninteracting replicas of the system. An important step in the proof consists of reducing the noncommuting case to the commuting one. This reduction relies on a result by Ogata (ref. 29, Theorem 1.1). A summary appears in Fig. 1.

*Set-up*. Let 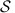 denote a system associated with a Hilbert space 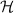, with a Hamiltonian *H*≡*Q*_0,_ and with observables (which we call ‘charges') *Q*_1_, *Q*_2_, …, *Q*_*c*_. The charges do not necessarily commute with each other: [*Q*_*j*_, *Q*_*k*_]≠0.

Consider *N* replicas of 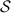, associated with the composite-system Hilbert space 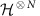. We average each charge *Q*_*j*_ over the *N* copies:


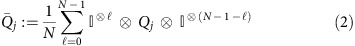


**Figure 2:**
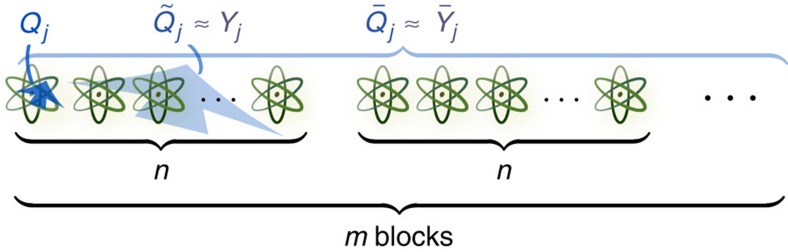
Noncommuting charges. We consider a thermodynamic system 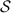 that has conserved charges *Q*_*j*_. These *Q*_*j*_'s might not commute with each other. The system occupies a thermal state whose form we derive. The derivation involves an approximate microcanonical state of a large system that contains the system of interest. Consider a block of *n* copies of 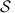. Most copies act, jointly, similarly to a bath for the copy of interest. We define 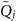 as the average of the *Q*_*j*_'s of the copies in the block. Applying results from Ogata^29^, we find operators 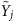 that are close to the 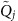
's and that commute with each other. Next, we consider *m* such blocks. This set of *m* blocks contains *N*=*mn* copies of 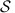. Averaging the 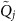
's over the blocks, for a fixed *j*-value, yields a global observable 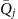. The 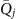
's are approximated by 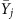
's. The 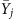
's are the corresponding averages of the 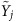
's. The approximate global charges 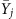 commute with each other. The commuting 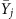
's enable us to extend the concept of a microcanonical ensemble from the well-known context in which all charges commute to truly quantum systems whose charges do not necessarily commute.

The basic idea is that, as *N* grows, the averaged operators 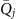 come increasingly to commute. Indeed, there exist operators 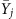 that commute with each other and that approximate the averages (ref. 29, Theorem 1.1). An illustration appears in Fig. 2.

*Derivation*. Since the 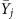
's commute mutually, they can be measured simultaneously. More importantly, the joint Hilbert space 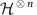 contains a subspace on which each 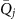 has prescribed values close to *v*_*j*_. Let 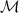 denote the subspace. Perhaps unsurprisingly, because the 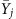
's approximate the 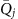
's, each state in 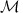 has a nearly well-defined value of 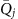 near *v*_*j*_. If 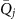 is measured, the distribution is sharply peaked around *v*_*j*_. We can also show the opposite: every state with nearly well-defined values *v*_*j*_ of all 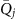
's has most of its probability weight in 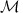.

These two properties show that 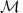 is an approximate microcanonical subspace for the 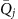
's with values *v*_*j*_. The notion of the approximate microcanonical subspace is the first major contribution of our work. It captures the idea that for large *N* we can approximately fix the values of the noncommuting charges *Q*_*j*_. An approximate microcanonical subspace 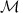 is any subspace consisting of the whole-system states whose average observables 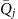 have nearly well-defined values *v*_*j*_. More precisely, a measurement of any 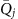 has a high probability of yielding a value near *v*_*j*_ if and only if most of the state's probability weight lies in 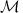.

Normalizing the projector onto 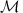 yields an approximate microcanonical ensemble, Ω. Tracing out every copy of 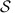 but the 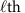 yields the reduced state 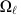. The distance between 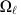 and the NATS *γ*_**v**_ can be quantified by the relative entropy


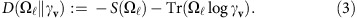


Here 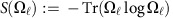 is the von Neumann entropy. The relative entropy *D* is bounded by the trace norm 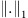, which quantifies the distinguishability of 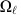 and *γ*_**v**_ (ref. 30):


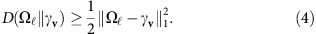


Our second main result is that if Ω is an approximate microcanonical ensemble, then the average, over systems 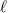, of the relative entropy *D* between 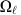 and *γ*_**v**_ is small:


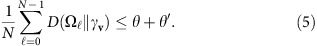


The parameter 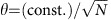 vanishes in the many-copy limit. *θ′* depends on the number *c* of charges, on the approximate expectation values *v*_*j*_, on the eigenvalues of the charges *Q*_*j*_ and on the (small) parameters in terms of which 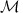 approximates a microcanonical subspace.

Inequality (5) capstones the derivation. The inequality follows from bounding each term in equation (3), the definition of the relative entropy *D*. The entropy 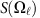 is bounded with *θ*. This bound relies on Schumacher's Theorem, which quantifies the size of a high-probability subspace like 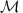 with an entropy *S*(*γ*_**v**_)^31^. We bound the second term in the *D* definition with *θ′*. This bound relies on the definition of 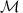: outcomes of measurements of the 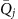
's are predictable up to parameters on which *θ′* depends.

Finally, we present conditions under which the approximate microcanonical subspace 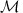 exists. Several parameters quantify the approximation. The parameters are shown to be interrelated and to approach zero simultaneously as *N* grows. In particular, the approximate microcanonical subspace 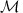 exists if *N* is great enough.

This microcanonical derivation offers a physical counterpoint to Jaynes's maximum-entropy derivation of the NATS's form. We relate the NATS to the physical picture of a small subsystem in a vast universe that occupies an approximate microcanonical state. This vast universe allows the correspondence principle to underpin our argument. In the many-copy limit as *N*→∞, the principle implies that quantum behaviours should vanish, as the averages of the noncommuting charges *Q*_*j*_ come to be approximated by commuting 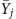
's. Drawing on Ogata's Theorem 1.1 (ref. 29), we link thermality in the presence of noncommutation to the physical correspondence principle.

### Dynamical considerations

The microcanonical and maximum-entropy arguments rely on kinematics and information theory. But we wish to associate the NATS with the fixed point of dynamics. The microcanonical argument, combined with canonical typicality, suggests that the NATS is the equilibrium state of typical dynamics. Canonical typicality enables us to model the universe's state with a pure state in the approximate microcanonical subspace 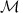. If a large system occupies a randomly chosen pure state, the reduced state of a small subsystem is close to thermal^5^,^6^,^7^,^8^.

Consider, as in the previous section, *N* copies of the system 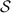. By Ω, we denoted the composite system's approximately microcanonical state. We denoted by 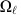 the reduced state of the 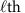 copy, formed by tracing out most copies from Ω. Imagine that the whole system occupies a pure state 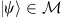. Denote by 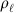 the reduced state of the 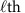 copy. 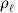 is close to 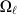, on average, by canonical typicality^7^:


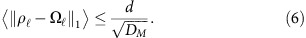


The average 〈.〉 is over pure states 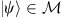. The trace norm is denoted by 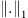; 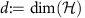 denotes the dimensionality of the Hilbert space 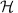 of one copy of 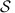; and 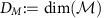 denotes the dimensionality of the approximate microcanonical subspace 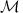.

We have bounded, using canonical typicality, the average trace norm between 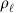 and 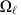. We can bound the average trace norm between 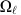 and the NATS *γ*_**v**_, using our microcanonical argument. (Supplementary equation (10) bounds the average relative entropy *D* between 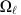 and *γ*_**v**_. Pinsker's inequality, Ineq. (4), lower bounds *D* in terms of the trace norm.) Combining these two trace-norm bounds via the triangle inequality, we bound the average distance between 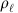 and *γ*_**v**_:


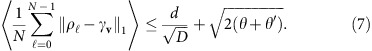


If the whole system occupies a random pure state 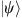 in 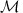, the reduced state 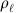 of a subsystem is, on average, close to the NATS *γ*_**v**_.

Sufficiently ergodic dynamics is expected to evolve the whole-system state to a 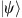 that satisfies Ineq. (7): suppose that the whole system begins in a pure state 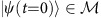. Suppose that the system's Hamiltonian commutes with the charges: [*H*, *Q*_*j*_]=0 for all *j*=1, …, *c*. The dynamics conserves the charges. Hence, most of the amplitude of 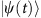 remains in 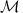 for appreciable times. Over sufficient times, ergodic dynamics yields a state 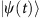 that can be regarded as random. Hence the reduced state is expected be close to 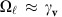 for most long-enough times *t*.

Exploring how the dynamics depends on the number of copies of the system offers promise for interesting future research.

### Resource theory

A thermodynamic resource theory is an explicit characterization of a thermodynamic system's resources, free states and free operations with a rigorous mathematical model. The resource theory specifies what an experimenter considers valuable (for example, work) and what is considered plentiful, or free (for example, thermal states). To define a resource theory, we specify allowed operations and which states can be accessed for free. We use this framework to quantify the resources needed to transform one state into another.

The first resource theory was entanglement theory^32^. The theory's free operations are local operations and classical communication. The free states are the states that can be easily prepared with local operations and classical communication, the separable states. Entangled states constitute valuable resources. One can quantify entanglement using this resource theory.

We present a resource theory for thermodynamic systems that have noncommuting conserved charges *Q*_*j*_. The theory is defined by its set of free operations, which we call ‘non-Abelian thermal operations' (NATO). NATO generalize thermal operations^15^,^18^. How to extend thermodynamic resource theories to conserved quantities other than energy was noted in refs 18, 20, 21. The NATO theory is related to the resource theory in ref. 27.

We supplement these earlier approaches with two additions. First, a battery has a work payoff function dependent on chemical potentials. We use this payoff function to define chemical work. Second, we consider a reference system for a non-Abelian group. The reference system is needed to resolve the difficulty encountered in refs 21, 27: There might be no nontrivial operations which respect all the conservation laws. The laws of physics require that any operation performed by an experimenter commutes with all the charges. If the charges fail to commute with each other, there might be no non-trivial unitaries that commute with all of them. In practice, one is not limited by such a stringent constraint. The reason is that an experimenter has access to a reference frame^33^,^34^,^35^.

A reference frame is a system *W* prepared in a state such that for any unitary on a system *S* which does not commute with the charges of *S*, some global unitary on *WS* conserves the total charges and approximates the unitary on *S* to arbitrary precision. The reference frame relaxes the strong constraint on the unitaries. The reference frame can be merged with the battery, in which the agent stores the ability to perform work. We refer to the composite as ‘the battery'. We denote its state by *ρ*_W_. The battery has a Hamiltonian *H*_W_ and charges *Q*_*j*_W__, described below.

Within this resource theory, the NATS emerges in two ways:

1. The NATS is the unique state from which work cannot be extracted, even if arbitrarily many copies are available. That is, the NATS is completely passive.
2. The NATS is the only state of *S* that remains invariant under the free operations during which no work is performed on *S*.

On proving the latter condition, we prove second laws for thermodynamics with noncommuting charges. These laws provide necessary conditions for a transition to be possible. In some cases, we show, the laws are sufficient. These second laws govern state transitions of a system *ρ*_S_, governed by a Hamiltonian *H*_S_, whose charges 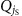 can be exchanged with the surroundings. We allow the experimenter to couple *ρ*_S_ to free states *ρ*_R_. The form of *ρ*_R_ is determined by the Hamiltonian *H*_R_ and the charges 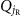 attributable to the free system. We will show that these free states have the form of the NATS. As noted above, no other state could be free. If other states were free, an arbitrarily large amount of work could be extracted from them.

Before presenting the second laws, we must define ‘work'. In textbook examples about gases, one defines work as *δW*=*p* d*V*, because a change in volume at a fixed pressure can be translated into the ordinary notion of mechanical work. If a polymer is stretched, then *δW*=*F* d*x*, wherein *x* denotes the polymer's linear displacement and *F* denotes the restoring force. If *B* denotes a magnetic field and *M* denotes a medium's magnetization, *δW*=*B* d*M*. The definition of ‘work' can depend on one's ability to transform changes in thermodynamic variables into a standard notion of ‘work,' such as mechanical or electrical work.

Our approach is to define a notion of chemical work. We could do so by modelling explicitly how the change in some quantity *Q*_*j*_ can be used to extract *μ*_*j*_ *δQ*_*j*_ work. Explicit modelling would involve adding a term to the battery Hamiltonian *H*_W_. Rather than considering a specific work Hamiltonian or model of chemical work, however, we consider a work payoff function,


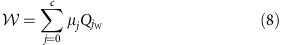


The physical situation could determine the form of this 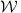. For example, the *μ*_*j*_'s could denote the battery's chemical potentials. In such a case, 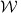 would denote the battery's total Hamiltonian, which would depend on those potentials.

We choose a route conceptually simpler than considering an explicit Hamiltonian and battery system, however. We consider equation (8) as a payoff function that defines the linear combination of charges that interests us. We define the (chemical) work expended or distilled during a transformation as the change in the quantum expectation value 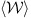.

The form of 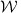 is implicitly determined by the battery's structure and by how charges can be converted into work. For our purposes, however, the origin of the form of 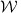 need not be known. 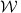 will uniquely determine the *μ*_*j*_'s in the NATS. Alternatively, we could first imagine that the agent could access, for free, a particular NATS. This NATS's form would determine the work function's form. If the charges commute, the corresponding Gibbs state is known to be the unique state that is completely passive with respect to the observable (8).

In Supplementary Note 3, we specify the resource theory for noncommuting charges in more detail. We show how to construct allowable operations, using the reference frame and battery. From the allowable operations, we derive a zeroth law of thermodynamics.

*Complete passivity and zeroth law*. This zeroth law relates to the principle of complete passivity, discussed in refs 25, 26. A state is complete passive if an agent cannot extract work from arbitrarily many copies of the state. In the resource theory for heat exchanges, completely passive states can be free. They do not render the theory trivial because no work can be drawn from them^17^.

In the NATO resource theory, we show, the only reasonable free states have the NATS's form. The free states' chemical potentials equal the *μ*_*j*_'s in the payoff function 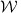, at some common fixed temperature. Any other state would render the resource theory trivial: from copies of any other state, arbitrarily much work could be extracted for free. Then, we show that the NATS is preserved by NATO, the operations that perform no work on the system.

The free states form an equivalence class. They lead to notions of temperature and chemical potentials *μ*_*j*_. This derivation of the free state's form extends complete passivity and the zeroth law from ref. 17 to noncommuting conserved charges. The derivation further solidifies the role of the NATS in thermodynamics.

*Second laws*. The free operations preserve the NATS. We therefore focus on contractive measures of states' distances from the NATS. Contractive functions decrease monotonically under the free operations. Monotones feature in ‘second laws' that signal whether NATO can implement a state transformation. For example, the α-Rényi relative entropies between a state and the NATS cannot increase.

Monotonicity allows us to define generalized free energies as


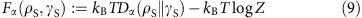


wherein *β*≡1/(*k*_B_*T*) and *k*_B_ denotes Boltzmann's constant. *γ*_S_ denotes the NATS with respect to the Hamiltonian *H*_S_ and the charges 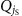 of the system *S*. The partition function is denoted by *Z*. Various classical and quantum definitions of the Rényi relative entropies *D*_*α*_ are known to be contractive^17^,^36^,^37^,^38^,^39^. The free energies *F*_*α*_ decrease monotonically if no work is performed on the system. Hence the *F*_*α*_'s characterize natural second laws that govern achievable transitions.

For example, the classical Rényi divergences 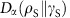 are defined as


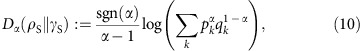


wherein *p*_*k*_ and *q*_*k*_ denote the probabilities of *ρ*_S_ and of *γ*_S_ in the 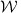 basis. The *D*_*α*_'s lead to second laws that hold even in the absence of a reference frame and even outside the context of the average work.

The *F*_*α*_'s reduce to the standard free energy when averages are taken over large numbers. Consider the asymptotic (‘thermodynamic') limit in which many copies 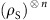 of *ρ*_S_ are transformed. Suppose that the agent has some arbitrarily small probability *ɛ* of failing to implement the desired transition. *ɛ* can be incorporated into the free energies via a technique called ‘smoothing'^17^. The average, over copies of the state, of every smoothed 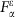 approaches *F*_1_ (ref. 17):


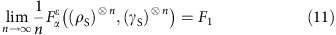



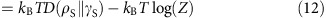



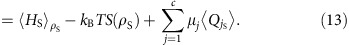


We have invoked the relative entropy's definition


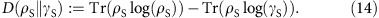


Note the similarity between the many-copy average *F*_1_ in equation (13) and the ordinary free energy, *F*=*E*−*TS*+∑_*j*_ *μ*_*j*_*N*_*j*_. The monotonic decrease of *F*_1_ constitutes a necessary and sufficient condition for a state transition to be possible in the presence of a reference system in the asymptotic limit.

In terms of the generalized free energies, we formulate second laws.

*Proposition 1*: in the presence of a heat bath of inverse temperature *β* and chemical potentials *μ*_*j*_, the free energies *F*_*α*_(*ρ*_S_, *γ*_S_) decrease monotonically:


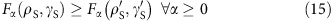


wherein *ρ*_S_ and 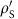 denote the system's initial and final states. The system's Hamiltonian and charges may transform from *H*_S_ and 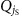 to 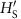 and 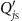. The NATSs associated with the same Hamiltonians and charges are denoted by *γ*_S_ and 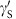. If


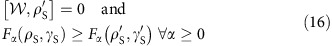


some NATO maps *ρ*_S_ to 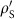.

As in ref. 17, additional laws can be defined in terms of quantum Rényi divergences^36^,^37^,^38^,^39^. This amounts to choosing, in Proposition 1, a definition of the Rényi divergence which accounts for the possibility that *ρ*_S_ and 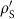 have coherences relative to the 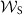 eigenbasis. Several measures are known to be contractive^36^,^37^,^38^,^39^. They, too, provide a new set of second laws.

*Extractable work*: in terms of the free energies *F*_*α*_, we can bound the work extractable from a resource state via NATO. We consider the battery *W* separately from the system *S* of interest. We assume that *W* and *S* occupy a product state. (This assumption is unnecessary if we focus on average work.) Let *ρ*_W_ and 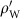 denote the battery's initial and final states.

For all *α*,


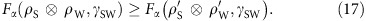


Since 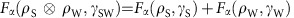,


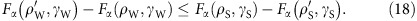


The left-hand side of Ineq. (18) represents the work extractable during one implementation of *ρ*_S_→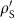. Hence the right-hand side bounds the work extractable during the transition.

Consider extracting work from many copies of *ρ*_S_ (that is, extracting work from 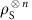) in each of many trials. Consider the average-over-trials extracted work, defined as 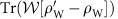. The average-over-trials work extracted per copy of *ρ*_S_ is 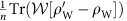. This average work per copy has a high probability of lying close to the change in the expectation value of the system's work function, 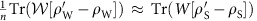, if *n* is large.

Averaging over the left-hand side of Ineq. (18) yields the average work 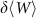 extracted per instance of the transformation. The average over the right-hand side approaches the change in *F*_1_ (equation (13)):


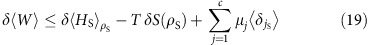


This bound is achievable with a reference system, as shown in refs 40, 41.

We have focused on the extraction of work defined by 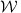. One can extract, instead, an individual charge *Q*_*j*_. The second laws do not restrict single-charge extraction. But extracting much of one charge *Q*_*j*_ precludes the extraction of much of another charge, *Q*_*k*_. In Supplementary Note 3, we discuss the trade-offs amongst extractions of different charges *Q*_*j*_.

## Discussion

We have derived, via multiple routes, the form of the thermal state of a system that has noncommuting conserved charges. First, we regarded the system as part of a vast composite that occupied an approximate microcanonical state. Tracing out the environment yields a reduced state that lies, on average, close to a thermal state of the expected form. This microcanonical argument, with canonical typicality, suggests that the NATS is the fixed point of typical dynamics. Defining a resource theory, we showed that the NATS is the only completely passive state and is the only state preserved by free operations. These physical derivations buttress Jaynes's information-theoretic derivation from the principle of maximum entropy.

Our derivations also establish tools applicable to quantum noncommutation in thermodynamics. In the microcanonical argument, we introduced an approximate microcanonical state Ω. This Ω resembles the microcanonical ensemble associated with a fixed energy, a fixed particle number and so on but accommodates noncommuting charges. Our complete-passivity argument relies on a little-explored resource theory for thermodynamics, in which free unitaries conserve noncommuting charges.

We expect that the equilibrium behaviours predicted here may be observed in experiments. Quantum gases have recently demonstrated equilibrium-like predictions about integrable quantum systems^11^,^13^.

From a conceptual perspective, our work shows that notions previously considered relevant only to commuting charges—for example, the microcanonical subspace—extend to noncommuting charges. This work opens fully quantum thermodynamics to analysis with familiar, but suitably adapted, technical tools.

### Data availability

Data sharing is not applicable to this article, as no data sets were generated or analysed during this study.

## Additional information

**How to cite this article:** Yunger Halpern, N. *et al*. Microcanonical and resource-theoretic derivations of the thermal state of a quantum system with noncommuting charges. *Nat. Commun.* 7:12051 doi: 10.1038/ncomms12051 (2016).

## Supplementary Material

Supplementary Information Supplementary Notes 1-3 and Supplementary References.
