# Supplementary material for: Microcanonical and resource-theoretic derivations of the thermal state of a quantum system with noncommuting charges
Source: Nat Commun. 2016 Jul 7;7:12051. doi: 10.1038/ncomms12051 (PMC4941045; doi:10.1038/ncomms12051)
Supplement: Supplementary Information — Supplementary Notes 1-3 and Supplementary References. [file ncomms12051-s1.pdf]

Supplementary Information

SUPPLEMENTARY NOTE 1: MICROCANONICAL DERIVATION OF THE NATS'S FORM

Upon describing the set-up, we will define an approximate microcanonical subspace $\mathcal{M}$. Normalizing the projector onto $\mathcal{M}$ yields an approximate microcanonical state Ω. Tracing out most of the system from Ω leads, on average, to a state close to the Non-Abelian Thermal State $\gamma_{\mathbf{v}}$. Finally, we derive conditions under which $\mathcal{M}$ exists.

Set-up: Consider a system $\mathcal{S}$ associated with a Hilbert space $\mathcal{H}$ of dimension $d := \dim(\mathcal{H})$. Let $H \equiv Q_0$ denote the Hamiltonian. We call observables denoted by $Q_1, \ldots, Q_c$ "charges." Without loss of generality, we assume that the Q_j's form a linearly independent set. The Q_j's do not necessarily commute with each other. They commute with the Hamiltonian if they satisfy a conservation law,

$$[H, Q_j] = 0 \;\; \forall j = 1, \ldots, c. \tag{1}$$

This conservation is relevant to dynamical evolution, during which the NATS may arise as the equilibrium state. However, our microcanonical derivation does not rely on conservation.

Bath, blocks, and approximations to charges: Consider many copies n of the system $\mathcal{S}$. Following Ogata [1], we consider an average $\tilde{Q}_j$, over the n copies, of each charge Q_j (Fig. 2 of the main text):

$$\tilde{Q}_j := \frac{1}{n} \sum_{\ell=0}^{n-1} \mathbb{I}^{\otimes \ell} \otimes Q_j \otimes \mathbb{I}^{\otimes (n-1-\ell)}. \tag{2}$$

In the large-n limit, the averages $\tilde{Q}_j$ are approximated by observables $\tilde{Y}_j$ that commute [1, Theorem 1.1]:

$$\|\tilde{Q}_j - \tilde{Y}_j\|_\infty \leq \epsilon_{\mathrm{O}}(n) \to 0, \text{ and} \tag{3}$$

$$[\tilde{Y}_j, \tilde{Y}_k] = 0 \;\; \forall j, k = 0, \ldots, c. \tag{4}$$

The $\tilde{Y}_j$'s are defined on $\mathcal{H}^{\otimes n}$, $\| \cdot \|_\infty$ denotes the operator norm, and $\epsilon_{\mathrm{O}}(n)$ denotes a function that approaches zero as $n \to \infty$.

Consider m blocks of n copies of $\mathcal{S}$, i.e., $N = nm$ copies of $\mathcal{S}$. We can view one copy as the system of interest and $N-1$ copies as a bath. Consider the average, over N copies, of a charge Q_j:

$$\bar{Q}_j := \frac{1}{N} \sum_{\ell=0}^{N-1} \mathbb{I}^{\otimes \ell} \otimes Q_j \otimes \mathbb{I}^{\otimes (N-1-\ell)}. \tag{5}$$

This $\bar{Q}_j$ equals also the average, over m blocks, of the block average $\tilde{Q}_j$:

$$\bar{Q}_j = \frac{1}{m} \sum_{\lambda=0}^{m-1} \mathbb{I}^{\otimes \lambda n} \otimes \tilde{Q}_j \otimes \mathbb{I}^{\otimes [N - n(\lambda+1)]}. \tag{6}$$

Let us construct observables $\bar{Y}_j$ that approximate the $\bar{Q}_j$'s and that commute: $[\bar{Y}_j, \bar{Y}_k] = 0$, and $\|\bar{Q}_j - \bar{Y}_j\|_\infty \leq \epsilon$ for all m. Since $\tilde{Y}_j$ approximates the $\tilde{Q}_j$ in Eq. (6), we may take

$$\bar{Y}_j = \frac{1}{m} \sum_{\lambda=0}^{m-1} \mathbb{I}^{\otimes \lambda n} \otimes \tilde{Y}_j \otimes \mathbb{I}^{\otimes [N - n(\lambda+1)]}. \tag{7}$$

Approximate microcanonical subspace: Recall the textbook derivation of the form of the thermal state of a system that exchanges commuting charges with a bath. The composite system's state occupies a microcanonical subspace. In every state in the subspace, every whole-system charge, including the energy, has a well-defined value. Charges that fail to commute might not have well-defined values simultaneously. But, if N is large, the $\bar{Q}_j$'s nearly commute; they can nearly have well-defined values simultaneously. This approximation motivates our definition of an approximate microcanonical subspace $\mathcal{M}$. If the composite system occupies any state in $\mathcal{M}$, one has a high probability of being able to predict the outcome of a measurement of any $\bar{Q}_j$.

Definition 1. *For $\eta, \eta', \epsilon, \delta, \delta' > 0$, an $(\epsilon, \eta, \eta', \delta, \delta')$-*approximate microcanonical (a.m.c.) subspace *$\mathcal{M}$ of $\mathcal{H}^{\otimes N}$ associated with observables $\bar{Q}_j$ and with approximate expectation values v_j consists of the states ω for which the probability distribution over the possible outcomes of a measurement of any $\bar{Q}_j$ peaks sharply about v_j. More precisely, we denote by Π_j^η the projector onto the direct sum of the eigensubspaces of $\bar{Q}_j$ associated with the eigenvalues in the interval $[v_j - \eta\Sigma(Q_j), v_j + \eta\Sigma(Q_j)]$. Here, $\Sigma(Q) = \lambda_{\max}(Q) - \lambda_{\min}(Q)$ is the spectral diameter of an observable Q. $\mathcal{M}$ must satisfy the following conditions:*

1. *Let ω denote any state, defined on $\mathcal{H}^{\otimes N}$, whose support lies in $\mathcal{M}$. A measurement of any $\bar{Q}_j$ is likely to yield a value near v_j:*

$$\mathrm{supp}(\omega) \subset \mathcal{M} \quad \Rightarrow \quad \mathsf{Tr}(\omega \Pi_j^\eta) \geq 1 - \delta \;\; \forall j. \tag{8}$$

2. *Conversely, consider any state ω, defined on $\mathcal{H}^{\otimes N}$, whose measurement statistics peak sharply. Most of the state's probability weight lies in $\mathcal{M}$:*

$$\mathsf{Tr}(\omega \Pi_j^{\eta'}) \geq 1 - \delta' \;\; \forall j \quad \Rightarrow \quad \mathsf{Tr}(\omega P) \geq 1 - \epsilon, \tag{9}$$

wherein P denotes the projector onto $\mathcal{M}$.

This definition merits two comments. First, $\mathcal{M}$ is the trivial (zero) subspace if the v_j's are inconsistent, i.e., if no state ρ satisfies $\mathsf{Tr}(\rho\, Q_j) = v_j \;\; \forall j$. Second, specifying $(\eta, \eta', \epsilon, \delta, \delta')$ does not specify a unique subspace. The inequalities enable multiple approximate microcanonical subspaces to satisfy Definition 1. The definition ensures, however, that any two such subspaces overlap substantially.

The approximate microcanonical subspace leads to the NATS: Let us show that Definition 1 exhibits the property desired of a microcanonical state: The reduced state of each subsystem is close to the NATS.

We denoted by P the projector onto the approximate microcanonical subspace $\mathcal{M}$. Normalizing the projector yields the approximate microcanonical state $\Omega := \frac{1}{\mathsf{Tr}(P)} P$. Tracing out all subsystems but the ℓth yields $\Omega_\ell := \mathsf{Tr}_{0,\ldots,\ell-1,\ell+1,\ldots,N-1}(\Omega)$.

We quantify the discrepancy between Ω_ℓ and the NATS with the relative entropy:

$$D(\Omega_\ell \| \gamma_{\mathbf{v}}) := -S(\Omega_\ell) - \mathsf{Tr}\left(\Omega_\ell \log(\gamma_{\mathbf{v}})\right). \tag{10}$$

wherein $S(\Omega_\ell) := -\mathsf{Tr}\left(\Omega_\ell \log(\Omega_\ell)\right)$ is the von Neumann entropy. The relative entropy is lower-bounded by the trace norm, which quantifies quantum states' distinguishability [2]:

$$D(\Omega_\ell \| \gamma_{\mathbf{v}}) \geq \frac{1}{2} \left\| \Omega_\ell - \gamma_{\mathbf{v}} \right\|_1^2 . \tag{11}$$

Theorem 2. *Let $\mathcal{M}$ denote an $(\epsilon, \eta, \eta', \delta, \delta')$-approximate microcanonical subspace of $\mathcal{H}^{\otimes N}$ associated with the Q_j's and the v_j's, for $N \geq [2 \left\| Q_j \right\|_\infty^2 / (\eta^2)] \log(2/\delta')$. The average, over the N subsystems, of the relative entropy between each subsystem's reduced state Ω_ℓ and the NATS is small:*

$$\frac{1}{N} \sum_{\ell=0}^{N-1} D(\Omega_\ell \| \gamma_{\mathbf{v}}) \leq \theta + \theta'. \tag{12}$$

This $\theta = (\text{const.})/\sqrt{N}$ is proportional to a constant dependent on ϵ, on the v_j's, and on d. This $\theta' = (c+1)(\text{const.})(\eta + 2\delta \cdot \max_j\{\left\| Q_j \right\|_\infty\})$ is proportional to a constant dependent on the v_j's.

Proof. We will bound each term in the definition (10) of the relative entropy D. The von Neumann-entropy term $S(\Omega_\ell)$, we bound with Schumacher's theorem for typical subspaces. The cross term is bounded, by the definition of the approximate microcanonical subspace $\mathcal{M}$, in terms of the small parameters that quantify the approximation.

First, we lower-bound the dimensionality of $\mathcal{M}$ in terms of $\epsilon, \eta, \eta', \delta$, and δ'. Imagine measuring some $\bar{Q}_j$ of the composite-system state $\gamma_{\mathbf{v}}^{\otimes N}$. This is equivalent to measuring each subsystem's Q_j, then averaging the outcomes. Each Q_j measurement would yield a random outcome $X_\ell^j \in [\lambda_{\min}(Q_j), \lambda_{\max}(Q_j)]$, for $\ell = 0, \ldots, N-1$. The average of these Q_j-measurement outcomes is tightly concentrated around v_j, by Hoeffding's Inequality [3]:

$$1 - \mathsf{Tr}\left(\gamma_{\mathbf{v}}^{\otimes N} \Pi_j^\eta\right) = \Pr\left\{ \left| \frac{1}{N} \sum_{\ell=0}^{N-1} X_\ell^j - v_j \right| > \eta\Sigma(Q_j) \right\} \tag{13}$$

$$\leq 2\exp\left(-2\eta^2 N\right) \tag{14}$$

$$\leq \delta', \tag{15}$$

for large enough N. From the second property in Definition 1, it follows that $\mathsf{Tr}\left(\gamma_{\mathbf{v}}^{\otimes N} P\right) \geq 1 - \epsilon$. Hence $\mathcal{M}$ is a high-probability subspace of $\gamma_{\mathbf{v}}^{\otimes N}$.

By Schumacher's Theorem, or by the stronger [4, Theorem I.19],

$$S(\Omega) = \log\Big(\dim(P)\Big) \geq NS\big(\gamma_{\mathbf{v}}\big) - (\text{const.})\sqrt{N} \tag{16}$$
$$= NS\big(\gamma_{\mathbf{v}}\big) - N\theta, \tag{17}$$

wherein $\theta := (\text{const.})/\sqrt{N}$. The constant depends on ϵ, d, and the charge values v_j. The entropy's subadditivity implies that $S(\Omega) \leq \sum_{\ell=0}^{N-1} S(\Omega_\ell)$. Combining this inequality with Ineq. (17) yields

$$S\big(\gamma_{\mathbf{v}}\big) - \theta \leq \frac{1}{N} \sum_{\ell=0}^{N-1} S(\Omega_\ell). \tag{18}$$

The support of Ω lies within $\mathcal{M}$: $\text{supp}(\Omega) \subset \mathcal{M}$. Hence $\mathsf{Tr}(\Omega\, \Pi_j^\eta) = 1 \geq 1 - \delta$ for all j. Let $\bar{\Omega} := \frac{1}{N}\sum_{\ell=0}^{N-1} \Omega_\ell$. We will bound the many-copy average

$$w_j := \mathsf{Tr}(Q_j\, \bar{\Omega}) = \frac{1}{N} \sum_{\ell=0}^{N-1} \mathsf{Tr}(\Omega_\ell\, Q_j) \tag{19}$$
$$= \mathsf{Tr}(\Omega\, \bar{Q}_j). \tag{20}$$

Let us bound this trace from both sides. Representing $\bar{Q}_j = \sum_q q\, \Pi_j^q$ in its eigendecomposition, we upper-bound the following average:

$$\mathsf{Tr}(\Omega\, \bar{Q}_j) = \sum_q q\, \mathsf{Tr}\left(\Omega\, \Pi_j^q\right) \tag{21}$$
$$\leq [v_j + \eta \Sigma(Q_j)]\, \mathsf{Tr}\left(\Omega\, \Pi_j^\eta\right) + \|Q_j\|_\infty \mathsf{Tr}\Big(\Omega\left[\mathbb{I} - \Pi_j^\eta\right]\Big) \tag{22}$$
$$\leq v_j + \|Q_j\|_\infty (\eta + \delta). \tag{23}$$

We complement this upper bound with a lower bound:

$$\mathsf{Tr}(\Omega\, \bar{Q}_j) \geq [v_j - \eta \Sigma(Q_j)]\, \mathsf{Tr}\left(\Omega\, \Pi_j^\eta\right) - \|Q_j\|_\infty \mathsf{Tr}\Big(\Omega\left[\mathbb{I} - \Pi_j^\eta\right]\Big) \tag{24}$$
$$\geq [v_j - \eta \Sigma(Q_j)](1 - \delta) - \|Q_j\|_\infty \delta. \tag{25}$$

Inequalities (23) and (25) show that the whole-system average w_j is close to the single-copy average v_j:

$$\xi_j := |w_j - v_j| = \left|\mathsf{Tr}(\Omega\, \bar{Q}_j) - v_j\right| \tag{26}$$
$$\leq (\eta + 2\delta)\, \|Q_j\|_\infty. \tag{27}$$

Let us bound the average relative entropy. By definition,

$$\frac{1}{N} \sum_{\ell=0}^{N-1} D\left(\Omega_\ell \| \gamma_{\mathbf{v}}\right) = -\frac{1}{N} \sum_{\ell=0}^{N-1} \Big[S(\Omega_\ell) + \mathsf{Tr}\Big(\Omega_\ell \log(\gamma_{\mathbf{v}})\Big)\Big]. \tag{28}$$

Let us focus on the second term. First, we substitute in the form of $\gamma_{\mathbf{v}}$ from Eq. (1) of the main text. Next, we substitute in for w_j, using Eq. (19). Third, we substitute in ξ_j, using Eq. (26). Fourth, we invoke the definition of

$S(\gamma_{\mathbf{v}})$, which we bound with Ineq. (18):

$$-\frac{1}{N}\sum_{\ell=0}^{N-1} \mathsf{Tr}\left(\Omega_\ell \log(\gamma_{\mathbf{v}})\right) \tag{29}$$

$$= \frac{1}{N}\sum_{\ell=0}^{N-1}\left[\log(Z) + \sum_{j=0}^{c} \mu_j \,\mathsf{Tr}(\Omega_\ell \, Q_j)\right] \tag{30}$$

$$= \log Z + \sum_{j=0}^{c} \mu_j w_j \tag{31}$$

$$\leq \log Z + \sum_{j=0}^{c} \mu_j v_j + \sum_{j=0}^{c} |\mu_j| \, \xi_j \tag{32}$$

$$= S(\gamma_{\mathbf{v}}) + \sum_{j=0}^{c} |\mu_j| \, \xi_j \tag{33}$$

$$\leq \frac{1}{N}\sum_{\ell=0}^{N-1} S(\Omega_\ell) + \theta + \sum_{j=0}^{c} |\mu_j| \, \xi_j . \tag{34}$$

Combining this inequality with Eq. (28) yields

$$\frac{1}{N}\sum_{\ell=0}^{N-1} D\left(\Omega_\ell \| \gamma_{\mathbf{v}}\right) \leq \theta + \sum_{j=0}^{c} |\mu_j| \, \xi_j \tag{35}$$

$$\leq \theta + (c+1)\left(\max_j |\mu_j|\right)\left(\max_j \xi_j\right) \tag{36}$$

$$\leq \theta + (c+1)\left(\max_j |\mu_j|\right)\left[(\eta + 2\delta)\cdot \max_j \left\{\|Q_j\|_\infty\right\}\right]. \tag{37}$$

The final inequality follows from Ineq. (27). Since the v_j's determine the μ_j-values, $(c+1)\left(\max_j |\mu_j|\right)$ is a constant determined by the v_j's. The final term in Ineq. (37), therefore, is upper-bounded by $\theta' = (c+1)(\text{const.})(\eta + 2\delta) \cdot \max_j \left\{\|Q_j\|_\infty\right\}$. □

Existence of an approximate microcanonical subspace: Definition 1 does not reveal under what conditions an approximate microcanonical subspace $\mathcal{M}$ exists. We will show that an $\mathcal{M}$ exists for $\epsilon, \eta, \eta', \delta, \delta'$ that can approach zero simultaneously, for sufficiently large N. First, we prove the existence of a microcanonical subspace for commuting observables. Applying this lemma to the $\tilde{Y}_j$'s shows that $\mathcal{M}$ exists for noncommuting observables.

Lemma 3. *Consider a Hilbert space $\mathcal{K}$ with commuting observables X_j, $j = 0, \ldots, c$. For all $\epsilon, \eta, \delta > 0$ and for sufficiently large m, there exists an $\left(\epsilon, \eta, \eta'{=}\eta, \delta, \delta'{=}\frac{\epsilon}{c+1}\right)$-approximate microcanonical subspace $\mathcal{M}$ of $\mathcal{K}^{\otimes m}$ associated with the observables X_j and with the approximate expectation values v_j.*

Proof. Recall that

$$\bar{X}_j = \frac{1}{m}\sum_{\lambda=0}^{m-1} \mathbb{I}^{\otimes \lambda} \otimes X_j \otimes \mathbb{I}^{\otimes(m-1-\lambda)} \tag{38}$$

is the average of X_j over the m subsystems. Denote by

$$\Xi_j^\eta := \left\{v_j - \eta \leq \bar{X}_j \leq v_j + \eta\right\} \tag{39}$$

the projector onto the direct sum of the $\bar{X}_j$ eigenspaces associated with the eigenvalues in $[v_j - \eta, v_j + \eta]$. Consider the subspace $\mathcal{M}_{\text{com}}^\eta$ projected onto by all the X_j's. The projector onto $\mathcal{M}_{\text{com}}^\eta$ is

$$P_{\text{com}} := \Xi_0^\eta \, \Xi_1^\eta \cdots \Xi_c^\eta . \tag{40}$$

Denote by ω any state whose support lies in $\mathcal{M}^{\eta}_{\text{com}}$. Let us show that ω satisfies the inequality in (8). By the definition of P_{com}, $\text{supp}(\omega) \subset \text{supp}(\Xi^{\eta}_{j})$. Hence $\mathsf{Tr}\left(\omega \Xi^{\eta}_{j}\right) = 1 \geq 1 - \delta$.

Let us verify the second condition in Definition 1. Consider any eigenvalue $\bar{y}_j$ of $\bar{Y}_j$, for each j. Consider the joint eigensubspace, shared by the $\bar{Y}_j$'s, associated with any eigenvalue $\bar{y}_1$ of $\bar{Y}_1$, with any eigenvalue $\bar{y}_2$ of $\bar{Y}_2$, etc. Denote the projector onto this eigensubspace of $\mathcal{H}^{\otimes N}$ by $\mathcal{P}_{\bar{y}_1,\cdots,\bar{y}_c}$.

Let $\delta' = \frac{\epsilon}{c+1}$. Let ω denote any state, defined on $\mathcal{H}^{\otimes N}$, for which $\mathsf{Tr}\left(\omega\, \Xi^{\eta}_{j}\right) \geq 1-\delta'$, for all $j = 0, \ldots, c$. The left-hand side of the second inequality in (9) reads, $\mathsf{Tr}\left(\omega P_{\text{com}}\right)$. We insert the resolution of identity $\sum_{\bar{y}_0,\ldots,\bar{y}_c} \mathcal{P}_{\bar{y}_0\ldots\bar{y}_c}$ into the trace. The property $\mathcal{P}^2 = \mathcal{P}$ of any projector $\mathcal{P}$ enables us to square each projector. Because $[\mathcal{P}_{\bar{y}_0\ldots\bar{y}_c}, P_{\text{com}}] = 0$,

$$\mathsf{Tr}\left(\omega P_{\text{com}}\right) = \mathsf{Tr}\left(\sum_{\bar{y}_0,\ldots,\bar{y}_c} \mathcal{P}_{\bar{y}_0\ldots\bar{y}_c} \omega \mathcal{P}_{\bar{y}_0\ldots\bar{y}_c} P_{\text{com}}\right) \tag{41}$$

$$=: \mathsf{Tr}\left(\omega' P_{\text{com}}\right), \tag{42}$$

wherein $\omega' := \sum_{\bar{y}_0,\ldots,\bar{y}_c} \mathcal{P}_{\bar{y}_0\ldots\bar{y}_c} \omega \mathcal{P}_{\bar{y}_0\ldots\bar{y}_c}$ is ω pinched with the complete set $\{\mathcal{P}_{\bar{y}_0\bar{y}_1\ldots\bar{y}_c}\}$ of projectors [5]. By this definition of ω', $\mathsf{Tr}\left(\omega'\, \Xi^{\eta}_{j}\right) = \mathsf{Tr}\left(\omega\, \Xi^{\eta}_{j}\right) \geq 1 - \delta'$, and $[\omega', \Xi^{\eta}_{j}] = 0$. For all j, therefore,

$$\omega'\, \Xi^{\eta}_{j} = \omega' - \omega' \left(\mathbb{I} - \Xi^{\eta}_{j}\right) =: \omega' - \Delta_j, \tag{43}$$

wherein

$$\mathsf{Tr}(\Delta_j) = \mathsf{Tr}\left(\omega' \left[\mathbb{I} - \Xi^{\eta}_{j}\right]\right) \leq \delta'. \tag{44}$$

Hence

$$\mathsf{Tr}\left(\omega' P_{\text{com}}\right) = \mathsf{Tr}\left(\omega'\, \Xi^{\eta}_{0}\, \Xi^{\eta}_{1} \cdots \Xi^{\eta}_{c}\right) \tag{45}$$

$$\geq \mathsf{Tr}\left(\left[\omega' - \Delta_0\right] \Xi^{\eta}_{1} \cdots \Xi^{\eta}_{c}\right) \tag{46}$$

$$\geq \mathsf{Tr}\left(\omega'\, \Xi^{\eta}_{1} \cdots \Xi^{\eta}_{c}\right) - \delta' \tag{47}$$

$$\geq \mathsf{Tr}\left(\omega'\right) - (c+1)\delta' \tag{48}$$

$$= 1 - (c+1)\delta' = 1 - \epsilon. \tag{49}$$

As ω satisfies (9), $\mathcal{M}^{\eta}_{\text{com}}$ is an $(\epsilon, \eta, \eta'{=}\eta, \delta, \delta'{=}\frac{\epsilon}{c+1})$-approximate microcanonical subspace. □

Lemma 3 proves the existence of an approximate microcanonical subspace $\mathcal{M}^{\eta}_{\text{com}}$ for the $\tilde{Y}_j$'s defined on $\mathcal{K} = \mathcal{H}^{\otimes n}$ and for sufficiently large n. In the subsequent discussion, we denote by Υ^{η}_{j} the projector onto the direct sum of the $\bar{Y}_j$ eigenspaces associated with the eigenvalues in $[v_j - \eta\Sigma(\tilde{Y}_j), v_j + \eta\Sigma(\tilde{Y}_j)]$. Passing from $\tilde{Y}_j$ to $\tilde{Q}_j$ to Q_j, we now prove that the same $\mathcal{M}^{\eta}_{\text{com}}$ is an approximate microcanonical subspace for the Q_j's.

Theorem 4. *Under the above assumptions, for every $\epsilon > (c+1)\delta' > 0$, $\eta > \eta' > 0$, $\delta > 0$, and all sufficiently large N, there exists an $(\epsilon, \eta, \eta', \delta, \delta')$-approximate microcanonical subspace $\mathcal{M}$ of $\mathcal{H}^{\otimes N}$ associated with the observables Q_j and with the approximate expectation values v_j.*

Proof. Let $\hat{\eta} = (\eta + \eta')/2$. For a constant $C_{\text{AP}} > 0$ to be determined later, let n be such that $\epsilon_{\text{O}} = \epsilon_{\text{O}}(n)$ from Ogata's result [1, Theorem 1.1] is small enough so that $\eta > \hat{\eta} + C_{\text{AP}}\epsilon_{\text{O}}^{1/3}$ and $\eta' < \hat{\eta} - C_{\text{AP}}\epsilon_{\text{O}}^{1/3}$, as well as such that $\hat{\delta} = \delta - C_{\text{AP}}\epsilon_{\text{O}}^{1/3} > 0$ and such that $\hat{\delta}' = \delta' + C_{\text{AP}}\epsilon_{\text{O}}^{1/3} \leq \frac{\epsilon}{c+1}$.

Choose m in Lemma 3 large enough such that an $(\epsilon, \hat{\eta}, \hat{\eta}'{=}\hat{\eta}, \hat{\delta}, \hat{\delta}')$-approximate microcanonical subspace $\mathcal{M} := \mathcal{M}_{\text{com}}$ associated with the commuting $\tilde{Y}_j$ exists, with approximate expectation values v_j.

Let ω denote a state defined on $\mathcal{H}^{\otimes N}$. We will show that, if measuring the $\bar{Y}_j$'s of ω yields sharply peaked statistics, measuring the $\bar{Q}_j$'s yields sharply peaked statistics. Later, we will prove the reverse (that sharply peaked $\bar{Q}_j$ statistics imply sharply peaked $\bar{Y}_j$ statistics).

Recall from Definition 1 that Π^{η}_{j} denotes the projector onto the direct sum of the $\bar{Q}_j$ eigenstates associated with the eigenvalues in $[v_j - \eta\Sigma(Q_j),\ v_j + \eta\Sigma(Q_j)]$. These eigenprojectors are discontinuous functions of the observables. Hence we look for better-behaved functions. We will approximate the action of Π^{η}_{j} by using

$$f_{\eta_0,\eta_1}(x) := \begin{cases} 1, & x \in [-\eta_0, \eta_0] \\ 0, & |x| > \eta_1 \end{cases}, \tag{50}$$

for $\eta_1 > \eta_0 > 0$. The Lipschitz constant of f is bounded by $\lambda := \frac{1}{\eta_1 - \eta_0} \in \mathbb{R}$.

The operator $f_{\eta_0 \Sigma(Q_j), \eta_1 \Sigma(Q_j)}(\bar{Q}_j - v_j \mathbb{I})$ approximates the projector $\Pi_j^{\eta_0}$. Indeed, as a matrix, $f_{\eta_0 \Sigma(Q_j), \eta_1 \Sigma(Q_j)}(\bar{Q}_j - v_j \mathbb{I})$ is sandwiched between the projector $\Pi_j^{\eta_0}$, associated with a width-η_0 interval around v_j, and a projector $\Pi_j^{\eta_1}$ associated with a width-η_1 interval of eigenvalues. $f_{\eta,\eta}$ is the indicator function on the interval $[-\eta, \eta]$. Hence $\Pi_j^{\eta} = f_{\eta \Sigma(Q_j), \eta \Sigma(Q_j)}(\bar{Q}_j - v_j \mathbb{I})$. Similarly, we can regard $f_{\eta_0 \Sigma(Q_j), \eta_1 \Sigma(Q_j)}(\bar{Y}_j - v_j \mathbb{I})$ as sandwiched between $\Upsilon_j^{\eta_0}$ and $\Upsilon_j^{\eta_1}$.

Because $\bar{Q}_j$ is close to $\bar{Y}_j$, $f(\bar{Q}_j)$ is close to $f(\bar{Y}_j)$: Let n be large enough so that, by [1, Theorem 1.1], $\|\bar{Q}_j - \bar{Y}_j\|_\infty \leq \epsilon_{\rm O}$. By [6, Theorem 4.1],

$$\|f_{\eta_0 \Sigma(Q_j), \eta_1 \Sigma(Q_j)}(\bar{Y}_j - v_j \mathbb{I}) - f_{\eta_0 \Sigma(Q_j), \eta_1 \Sigma(Q_j)}(\bar{Q}_j - v_j \mathbb{I})\|_\infty \leq \kappa_\lambda, \quad (51)$$

wherein $\kappa_\lambda = C_{\rm AP} \lambda \epsilon_{\rm O}^{2/3}$ and $C_{\rm AP}$ denotes a universal constant. Inequality (51) holds because f is λ-Lipschitz and bounded, so the Hölder norm in [6, Theorem 4.1] is proportional to λ.

Let us show that, if measuring the $\bar{Y}_j$'s of ω yields sharply peaked statistics, then measuring the $\bar{Q}_j$'s yields sharply peaked statistics, and vice versa. First, we choose $\eta_0 = \eta$, $\eta_1 = \eta + \epsilon_{\rm O}^{1/3}$, and $\lambda = \epsilon_{\rm O}^{-1/3}$ such that $\kappa := \kappa_\lambda = C_{\rm AP} \epsilon_{\rm O}^{1/3}$. By the "sandwiching,"

$$\mathsf{Tr}\left(\omega \Pi_j^{\eta + \epsilon_{\rm O}^{1/3}}\right) \geq \mathsf{Tr}\left(\omega f_{\eta_0 \Sigma(Q_j), \eta_1 \Sigma(Q_j)}\left[\bar{Q}_j - v_j \mathbb{I}\right]\right). \quad (52)$$

To bound the right-hand side, we invoke Ineq. (51):

$$\begin{aligned} \kappa &\geq \|f_{\eta_0 \Sigma(Q_j), \eta_1 \Sigma(Q_j)}(\bar{Y}_j - v_j \mathbb{I}) \\ &\quad - f_{\eta_0 \Sigma(Q_j), \eta_1 \Sigma(Q_j)}(\bar{Q}_j - v_j \mathbb{I})\|_\infty & (53) \\ &\geq \mathsf{Tr}\Big(f_{\eta_0 \Sigma(Q_j), \eta_1 \Sigma(Q_j)}(\bar{Y}_j - v_j \mathbb{I}) \\ &\quad - f_{\eta_0 \Sigma(Q_j), \eta_1 \Sigma(Q_j)}(\bar{Q}_j - v_j \mathbb{I})\Big) & (54) \\ &\geq \mathsf{Tr}\Big(\omega\Big[f_{\eta_0 \Sigma(Q_j), \eta_1 \Sigma(Q_j)}(\bar{Y}_j - v_j \mathbb{I}) \\ &\quad - f_{\eta_0 \Sigma(Q_j), \eta_1 v}(\bar{Q}_j - v_j \mathbb{I})\Big]\Big). & (55) \end{aligned}$$

Upon invoking the trace's linearity, we rearrange terms:

$$\begin{aligned} &\mathsf{Tr}\Big(\omega f_{\eta_0 \Sigma(Q_j), \eta_1 \Sigma(Q_j)}(\bar{Q}_j - v_j \mathbb{I})\Big) & (56) \\ &\quad \geq \mathsf{Tr}\Big(\omega f_{\eta_0 \Sigma(Q_j), \eta_1 \Sigma(Q_j)}(\bar{Y}_j - v_j \mathbb{I})\Big) - \kappa & (57) \\ &\quad \geq \mathsf{Tr}\left(\omega \Upsilon_j^{\eta}\right) - \kappa. & (58) \end{aligned}$$

The final inequality follows from the "sandwiching" property of f_{η_0,η_1}. Combining Ineqs. (52) and (58) yields a bound on fluctuations in $\bar{Q}_j$ measurement statistics in terms of fluctuations in $\bar{Y}_j$ statistics:

$$\mathsf{Tr}\left(\omega\, \Pi_j^{\eta + \epsilon_{\rm O}^{1/3}}\right) \geq \mathsf{Tr}\left(\omega \Upsilon_j^{\eta}\right) - \kappa. \quad (59)$$

Now, we bound fluctuations in $\bar{Y}_j$ statistics with fluctuations in $\bar{Q}_j$ statistics. If $\eta_0 = \eta - \epsilon_{\rm O}^{1/3}$; $\eta_1 = \eta$; $\lambda = \epsilon_{\rm O}^{-1/3}$, as before, and $\kappa = \kappa_\lambda = C_{\rm AP} \epsilon_{\rm O}^{1/3}$, then

$$\mathsf{Tr}\left(\omega \Upsilon_j^{\eta}\right) \geq \mathsf{Tr}\left(\omega \Pi_j^{\eta - \epsilon_{\rm O}^{1/3}}\right) - \kappa. \quad (60)$$

Using Ineqs. (59) and (60), we can now show that $\mathcal{M} := \mathcal{M}_{\rm com}^{\hat{\eta}}$ is an approximate microcanonical subspace for the observables Q_j and the approximate charge values v_j. In other words, $\mathcal{M}$ is an approximate microcanonical subspace for the observables $\tilde{Q}_j$.

First, we show that $\mathcal{M}$ satisfies the first condition in Definition 1. Recall that $\mathcal{M}^{\eta}_{\text{com}}$ is an $\left(\epsilon, \eta, \eta'{=}\eta, \delta, \delta'{=}\frac{\epsilon}{c}\right)$-approximate microcanonical subspace for the observables $\tilde{Y}_j$ with the approximate charge values v_j, for all $\epsilon, \eta, \delta > 0$ and for large enough m (Lemma 3). Recall that $N = nm$. Choose $\delta = \hat{\delta} - \kappa > 0$. Let ω denote any state, defined on $\mathcal{H}^{\otimes N}$, whose support lies in $\mathcal{M} = \mathcal{M}^{\eta}_{\text{com}}$. Let $\hat{\eta} = \eta + \epsilon_{\text{O}}^{1/3}$. By the definitions of ω and $\mathcal{M}$, $\mathsf{Tr}\left(\omega \Upsilon^{\eta}_j\right) = 1 \geq 1 - \delta$. By Ineq. (59), therefore,

$$\mathsf{Tr}\left(\omega \Pi^{\hat{\eta}}_j\right) \geq \mathsf{Tr}\left(\omega \Upsilon^{\eta}_j\right) - \kappa \geq 1 - \delta - \kappa = 1 - \hat{\delta}. \tag{61}$$

Hence $\mathcal{M}$ satisfies Condition 1 in Definition 1.

To show that $\mathcal{M}$ satisfies Condition 2, let $\hat{\eta}' = \eta - \epsilon_{\text{O}}^{1/3}$, and let $\hat{\delta}' = \delta' - \kappa = \frac{\epsilon}{c} - C_{\text{AP}} \epsilon_{\text{O}}^{1/3} > 0$. Let ω in $\mathcal{H}^{\otimes N}$ satisfy $\mathsf{Tr}\left(\omega \Pi^{\hat{\eta}'}_j\right) \geq 1 - \hat{\delta}'$ for all j. By Ineq. (60),

$$\mathsf{Tr}\left(\omega \Upsilon^{\eta}_j\right) \geq 1 - \hat{\delta}' - \kappa = 1 - \delta'. \tag{62}$$

By Condition 2 in the definition of $\mathcal{M}^{\eta}_{\text{com}}$, therefore, at least fraction $1 - \epsilon$ of the probability weight of ω lies in $\mathcal{M}^{\eta}_{\text{com}} = \mathcal{M}$: $\mathsf{Tr}\left(\omega P_{\text{com}}\right) \geq 1 - \epsilon$. As $\mathcal{M}$ satisfies Condition 2, $\mathcal{M}$ is an $(\epsilon, \hat{\eta}, \hat{\eta}', \hat{\delta}, \hat{\delta}')$-approximate microcanonical subspace. □

This derivation confirms physically the information-theoretic maximum-entropy derivation. By "physically," we mean, "involving the microcanonical form of a composite system's state and from the tracing out of an environment." The noncommutation of the charges Q_j required us to define an approximate microcanonical subspace $\mathcal{M}$. The proof of the subspace's existence, under appropriate conditions, crowns the derivation.

The physical principle underlying this derivation is, roughly, the Correspondence Principle. The Q_j's of one copy of the system $\mathcal{S}$ fail to commute with each other. This noncommutation constitutes quantum mechanical behavior. In the many-copy limit, however, averages $\bar{Q}_j$ of the Q_j's are approximated by commuting $\bar{Y}_j$'s, whose existence was proved by Ogata [1]. In the many-copy limit, the noncommuting (quantum) problem reduces approximately to the commuting (classical) problem.

We stress that the approximate microcanonical subspace $\mathcal{M}$ corresponds to a set of observables Q_j and a set of values v_j. Consider the subspace $\mathcal{M}'$ associated with a subset of the Q_j's and their v_j's. This $\mathcal{M}'$ differs from $\mathcal{M}$. Indeed, $\mathcal{M}'$ typically has a greater dimensionality than $\mathcal{M}$, because fewer equations constrain it. Furthermore, consider a linear combination $Q' = \sum_{j=0}^{c} \mu_j Q_j$. The average $\bar{Q}'$ of N copies of Q' equals $\sum_{j=0}^{c} \mu_j \bar{Q}_j$. The approximate microcanonical subspace $\mathcal{M}$ of the whole set of Q_j's has the property that all states that lie mostly on it have sharply defined values near $v' = \sum_{j=0}^{c} \mu_j v_j$. Generally, however, our $\mathcal{M}$ is not an approximate microcanonical subspace for Q', or a selection of Q', Q'', etc., unless these primed operators span the same set of observables as the Q_j's.

SUPPLEMENTARY NOTE 2: DYNAMICAL CONSIDERATIONS

Inequality (7) of the main text is derived as follows: Let us focus on $\|\rho_\ell - \gamma_{\mathbf{v}}\|_1$. Adding and subtracting Ω_ℓ to the argument, then invoking the Triangle Inequality, yields

$$\|\rho_\ell - \gamma_{\mathbf{v}}\|_1 \leq \|\rho_\ell - \Omega_\ell\|_1 + \|\Omega_\ell - \gamma_{\mathbf{v}}\|_1. \tag{63}$$

We average over copies ℓ and average (via $\langle . \rangle$) over pure whole-system states $|\psi\rangle$. The first term on the right-hand side is bounded in Ineq. (6) of the main text:

$$\left\langle \frac{1}{N} \sum_{\ell=0}^{N-1} \|\rho_\ell - \gamma_{\mathbf{v}}\|_1 \right\rangle \leq \frac{d}{\sqrt{D_M}} + \left\langle \frac{1}{N} \sum_{\ell=0}^{N-1} \|\Omega_\ell - \gamma_{\mathbf{v}}\|_1 \right\rangle. \tag{64}$$

To bound the final term, we invoke Pinsker's Inequality [Ineq. (11)], $\|\Omega_\ell - \gamma_{\mathbf{v}}\|_1 \leq \sqrt{2D(\Omega_\ell || \gamma_{\mathbf{v}})}$. Averaging over ℓ and over states $|\psi\rangle$ yields

$$\left\langle \frac{1}{N} \sum_{\ell=0}^{N-1} \|\Omega_\ell - \gamma_{\mathbf{v}}\|_1 \right\rangle \leq \left\langle \frac{1}{N} \sum_{\ell=0}^{N-1} \sqrt{2D(\Omega_\ell || \gamma_{\mathbf{v}})} \right\rangle \tag{65}$$

$$\leq \left\langle \sqrt{\frac{2}{N} \sum_{\ell=0}^{N-1} D(\Omega_\ell || \gamma_{\mathbf{v}})} \right\rangle, \tag{66}$$

wherein D denotes the relative entropy. The second inequality follows from the square-root's concavity. Let us double each side of Ineq. (12), then take the square-root:

$$\sqrt{\frac{2}{N}\sum_{\ell=0}^{N-1} D(\Omega_\ell \| \gamma_{\mathbf{v}})} \leq \sqrt{2(\theta+\theta')}. \tag{67}$$

Combining the foregoing two inequalities, and substituting into Ineq. (64), yields Ineq. (7) of the main text.

SUPPLEMENTARY NOTE 3: DERIVATION FROM COMPLETE PASSIVITY AND RESOURCE THEORY

An alternative derivation of the thermal state's form relies on complete passivity. One cannot extract work from any number of copies of the thermal state via any energy-preserving unitary [7, 8]. We adapt this argument to noncommuting conserved charges. The Non-Abelian Thermal State is shown to be the completely passive "free" state in a thermodynamic resource theory.

Resource theories are models, developed in quantum information theory, for scarcity. Using a resource theory, one can calculate the value attributable to a quantum state by an agent limited to performing only certain operations, called "free operations." The first resource theory described pure bipartite entanglement [9]. Entanglement theory concerns how one can manipulate entanglement, if able to perform only local operations and classical communications. The entanglement theory's success led to resource theories for asymmetry [10], for stabilizer codes in quantum computation [11], for coherence [12], for quantum Shannon theory [13], and for thermodynamics, amongst other settings.

Resource-theoretic models for heat exchanges were constructed recently [14, 15]. The free operations, called "thermal operations," conserve energy. How to extend the theory to other conserved quantities was noted in [15]. The commuting-observables version of the theory was defined and analyzed in [16, 17], which posed questions about modeling noncommuting observables. We extend the resource theory to model thermodynamic exchanges of noncommuting observables. The free operations that define this theory, we term "Non-Abelian Thermal Operations" (NATO). This resource theory is related to that in [18]. We supplement earlier approaches with a work payoff function, as well as with a reference frame associated with a non-Abelian group.

This section is organized as follows. First, we introduce three subsystems and define work. Next, we define NATO. The NATO resource theory leads to the NATS via two routes:

1. The NATS is completely passive: The agent cannot extract work from any number of copies of $\gamma_{\mathbf{v}}$.

2. The NATS is the state preserved by NATO, the operations that require no work.

The latter condition leads to "second laws" for thermodynamics that involves noncommuting conserved charges. The second laws imply the maximum amount of work extractable from a transformation between states.

Subsystems: To specify a physical system in this resource theory, one specifies a Hilbert space, a density operator, a Hamiltonian, and operators that represent the system's charges. To specify the subsystem S of interest, for example, one specifies a Hilbert space $\mathcal{H}$; a density operator $\rho_{\rm S}$; a Hamiltonian $H_{\rm S}$; and charges $Q_{1_{\rm S}}, \ldots, Q_{c_{\rm S}}$.

Consider the group G formed from elements of the form $e^{i\boldsymbol{\mu}\cdot\mathbf{Q}}$. Each Q_j can be viewed as a generator. G is non-Abelian if the Q_j's fail to commute with each other. Following [19], we assume that G is a compact Lie group. The compactness assumption is satisfied if the system's Hilbert space is finite-dimensional. (We model the reference frame's Hilbert space as infinite-dimensional for convenience. Finite-size references can implement the desired protocols with arbitrary fidelity [19].)

We consider three systems, apart from S: First, R denotes a reservoir of free states. The resource theory is nontrivial, we prove, if and only if the free states have the NATS's form. Second, a battery W stores work. W doubles as a non-Abelian reference frame. Third, any other ancilla is denoted by A.

The Hamiltonian $H_{\rm tot} := H_{\rm S} + H_{\rm R} + H_{\rm W} + H_{\rm A}$ governs the whole system. The $j^{\rm th}$ whole-system charge has the form $Q_{j_{\rm tot}} := Q_{j_{\rm S}} + Q_{j_{\rm R}} + Q_{j_{\rm W}} + Q_{j_{\rm A}}$. Let us introduce each subsystem individually.

Battery: We define work by modeling the system that stores the work. In general, the mathematical expression for thermodynamic work depends on which physical degrees of freedom a system has. A textbook example concerns a gas, subject to a pressure p, whose volume increases by an amount dV. The gas performs an amount $dW = p\,dV$ of work. If a force F stretches a polymer through a displacement dx, $dW = -F\,dx$. If a material's magnetization decreases by an amount dM in the presence of a strength-B magnetic field, $dW = B\,dM$.

We model the ability to convert, into a standard form of work, a variation in some physical quantity. The model consists of an observable called a "payoff function." The payoff function is defined as

$$\mathcal{W} := \sum_{j=0}^{c} \mu_j Q_j \ . \tag{68}$$

We generally regard the payoff function as an observable of the battery's. We can also consider the $\mathcal{W}$ of the system of interest. If the system whose $\mathcal{W}$ we refer to is not obvious from context, we will use a subscript. For example, $\mathcal{W}_{\rm W}$ denotes the battery's work function.

One might assume that the battery exchanges only finite amounts of charges. Under this assumption, a realistically sized battery can implement the desired protocols with perfect fidelity [19].

Work: We define as average extracted work W the difference in expectation value of the payoff function $\mathcal{W}$:

$$W := \mathrm{Tr}\left(\rho'_{\rm W}\mathcal{W}\right) - \mathrm{Tr}\left(\rho_{\rm W}\mathcal{W}\right) \ . \tag{69}$$

The battery's initial and final states are denoted by $\rho_{\rm W}$ and $\rho'_{\rm W}$. If the expectation value increases, then $W > 0$, and work has been extracted from the system of interest. Otherwise, work has been expended.

We focus on the average work extracted in the asymptotic limit: We consider processing many copies of the system, then averaging over copies. Alternatively, one could focus on one instance of the transformation. The deterministic or maximal guaranteed work would quantify the protocol's efficiency better than the average work would [15, 20–22].

Reference frame: Reference frames have appeared in the thermodynamic resource theory for heat exchanges [23–25]. We introduce a non-Abelian reference frame into the thermodynamic resource theory for noncommuting conserved charges. Our agent's reference frame carries a representation of the G associated with the charges [10, 19].

The reference frame expands the set of allowed operations from a possibly trivial set. A superselection rule restricts the free operations, as detailed below. Every free unitary U conserves (commutes with) each charge. The system charges $Q_{j_{\rm S}}$ might not commute with each other. In the worst case, the $Q_{j_{\rm S}}$'s share no multidimensional eigensubspace. The only unitary that conserves all such $Q_{j_{\rm S}}$'s is trivial: $U \propto \mathbb{I}$.

A reference frame "frees up" dynamics, enabling the system to evolve nontrivially. A free unitary can fail to commute with a $Q_{j_{\rm S}}$ while preserving $Q_{j_{\rm tot}}$. This dynamics transfers charges between the system and the reference frame.

Our agent's reference frame doubles as the battery. The reference frame and battery are combined for simplicity, to reduce the number of subsystems under consideration.

Ancillas: The agent could manipulate extra subsystems, called "ancillas." A list $(\rho_{\rm A}, H_{\rm A}, Q_{1_{\rm A}}, \ldots, Q_{c_{\rm A}})$ specifies each ancilla A. Any ancillas evolve cyclically under free operations. That is, NATO preserve the ancillas' states, $\rho_{\rm A}$. If NATO evolved ancillas acyclically, the agent could "cheat," extracting work by degrading an ancilla [26].

Example ancillas include catalysts. A catalyst facilitates a transformation that could not occur for free in the catalyst's absence [26]. Suppose that a state $S = (\rho_{\rm S}, H_{\rm S}, Q_{1_{\rm S}}, \ldots, Q_{c_{\rm S}})$ cannot transform into a state $\tilde{S} = (\tilde{\rho}_{\rm S}, \tilde{H}_{\rm S}, \tilde{Q}_{1_{\rm S}}, \ldots, \tilde{Q}_{c_{\rm S}})$ by free operations: $S \not\mapsto \tilde{S}$. Some state $X = (\rho_{\rm X}, H_{\rm X}, Q_{1_{\rm X}}, \ldots, Q_{c_{\rm X}})$ might enable $S \otimes X \mapsto \tilde{S} \otimes X$ to occur for free. Such a facilitated transformation is called a "catalytic operation."

Non-Abelian Thermal Operations: NATO are the resource theory's free operations. NATO model exchanges of heat and of charges that might not commute with each other.

Definition 5. *Every Non-Abelian Thermal Operation (NATO) consists of the following three steps. Every sequence of three such steps forms a NATO:*

1. *Any number of free states* $(\rho_{\rm R}, H_{\rm R}, Q_{1_{\rm R}}, \ldots, Q_{c_{\rm R}})$ *can be added.*
2. *Any unitary* U *that satisfies the following conditions can be implemented on the whole system:*
   (a) U *preserves energy:* $[U, H_{\rm tot}] = 0$.
   (b) U *preserves every total charge:* $[U, Q_{j_{\rm tot}}] = 0 \ \forall j = 1, \ldots, c$.
   (c) *Any ancillas return to their original states:* $\mathrm{Tr}_{\setminus A}(U \rho_{\rm tot} U^\dagger) = \rho_{\rm A}$.
3. *Any subsystem can be discarded (traced out).*

Conditions 2a and 2b ensure that the energy and the charges are conserved. The allowed operations are G-invariant, or symmetric with respect to the non-Abelian group G. Conditions 2a and 2b do not significantly restrict the allowed operations, if the agent uses a reference frame. Suppose that the agent wishes to implement, on S, some unitary U that fails to commute with some $Q_{j_{\rm S}}$. U can be mapped to a whole-system unitary $\tilde{U}$ that conserves $Q_{j_{\rm tot}}$. The noncommutation represents the transfer of charges to the battery, associated with work.

The construction of $\tilde{U}$ from U is described in [19]. (We focus on the subset of free operations analyzed in [19].) Let $g, \phi \in G$ denote any elements of the symmetry group. Let T denote any subsystem (e.g., $T = S, W$). Let $V_{\rm T}(g)$ denote a representation, defined on the Hilbert space of system T, of g. Let $|\phi\rangle_{\rm T}$ denote a state of S that transforms as the left regular representation of G: $V_{\rm T}(g)|\phi\rangle_{\rm T} = |g\phi\rangle_{\rm T}$. U can be implemented on the system S of interest by the global unitary

$$\tilde{U} := \int d\phi \; |\phi\rangle\langle\phi|_{\rm W} \otimes [V_{\rm S}(\phi)\, U\, V_{\rm S}^{-1}(\phi)]. \tag{70}$$

The construction (70) does not increase the reference frame's entropy if the reference is initialized to $|\phi = 1\rangle_{\rm W}$. This nonincrease keeps the extracted work "clean" [22, 26, 27]. No entropy is "hidden" in the reference frame W. W allows us to implement the unitary U, providing or storing the charges consumed or outputted by the system of interest.

A. A zeroth law of thermodynamics: Complete passivity of the Non-Abelian Thermal State

Which states $\rho_{\rm R}$ should the resource-theory agent access for free? The free states are the only states from which work cannot be extracted via free operations. We will ignore S in this section, treating the reservoir R as the system of interest.

Free states in the resource theory for heat exchanges: Our argument about noncommuting charges will mirror the argument about extracting work when only the energy is conserved. Consider the thermodynamic resource theory for energy conservation. Let $H_{\rm R}$ denote the Hamiltonian of R. The free state $\rho_{\rm R}$ has the form $\rho_{\rm R} = e^{-\beta H_{\rm R}}/Z$ [16, 26]. This form follows from the canonical ensemble's completely passivity and from the nonexistence of any other completely passive state. Complete passivity was introduced in [7, 8].

Definition 6 (Passivity and complete passivity)**.** *Let ρ denote a state governed by a Hamiltonian H. ρ is passive with respect to H if no free unitary U can lower the energy expectation value of ρ:*

$$\nexists U \;:\; \mathsf{Tr}\left(U\rho U^\dagger H\right) < \mathsf{Tr}\left(\rho H\right). \tag{71}$$

That is, work cannot be extracted from ρ by any free unitary. If work cannot be extracted from any number n of copies of ρ, ρ is completely passive with respect to H:

$$\forall n = 1, 2, \ldots, \quad \nexists U \;:\; \mathsf{Tr}\left(U\rho^{\otimes n} U^\dagger H\right) < \mathsf{Tr}\left(\rho^{\otimes n} H\right). \tag{72}$$

A free U could lower the energy expectation value only if the energy expectation value of a work-storage system increased. This transfer of energy would amount to work extraction.

Conditions under which ρ is passive have been derived [7, 8]: Let $\{p_i\}$ and $\{E_i\}$ denote the eigenvalues of ρ and H. ρ is passive if

1. $[\rho, H] = 0$ and
2. $E_i > E_j$ implies that $p_i \leq p_j$ for all i, j.

One can check that $e^{-\beta H_{\rm R}}/Z$ is completely passive with respect to $H_{\rm R}$.

No other states are completely passive (apart from the ground state). Suppose that the agent could access copies of some $\rho_0 \neq e^{-\beta H_{\rm R}}/Z$. The agent could extract work via thermal operations [26]. Free (worthless) states could be transformed into a (valuable) resource for free. Such a transformation would be unphysical, rendering the resource theory trivial, in a sense. (As noted in [28], if a reference frame is not allowed, the theory might be nontrivial in that creating superpositions of energy eigenstates would not be possible).

Free states in the resource theory of Non-Abelian Thermal Operations: We have reviewed the free states in the resource theory for heat exchanges. Similar considerations characterize the resource theory for noncommuting charges Q_j. The free states, we show, have the NATS's form. If any other state were free, the agent could extract work for free.

Theorem 7. *There exists an $m > 0$ such that a NATO can extract a nonzero amount of chemical work from $(\rho_{\rm R})^{\otimes m}$ if and only if $\rho_{\rm R} \neq e^{-\beta\,(H_{\rm R} + \sum_j \mu_j Q_{j_{\rm R}})}/Z$ for some $\beta \in \mathbb{R}$.*

Proof. We borrow from [7, 8] the proof that canonical-type states, and only canonical-type states, are completely passive. We generalize complete passivity with respect to a Hamiltonian H to complete passivity with respect to the work function $\mathcal{W}$.

Every free unitary preserves every global charge. Hence the lowering of the expectation value of the work function $\mathcal{W}$ of a system amounts to transferring work from the system to the battery:

$$\Delta\,\mathsf{Tr}(\mathcal{W}_{\mathrm{W}}\rho_{\mathrm{W}}) = -\Delta\,\mathsf{Tr}(\mathcal{W}_{\mathrm{R}}\rho_{\mathrm{R}}). \tag{73}$$

Just as $e^{-\beta H}/Z$ is completely passive with respect to H [7, 8], the NATS is completely passive with respect to $\mathcal{W}_{\mathrm{R}}$ for some β.

Conversely, if ρ_{R} is not of the NATS form, it is not completely passive with respect to $\mathcal{W}_{\mathrm{R}}$. Some unitary $U_{\mathrm{R}^{\otimes m}}$ lowers the energy expectation value of $\rho_{\mathrm{R}}^{\otimes m}$, $\mathsf{Tr}(U_{\mathrm{R}^{\otimes m}}[\rho_{\mathrm{R}}^{\otimes m}]U_{\mathrm{R}^{\otimes m}}^{\dagger}\mathcal{W}_{\mathrm{R}^{\otimes m}}) < \mathsf{Tr}(\rho_{\mathrm{R}}^{\otimes m}\mathcal{W}_{\mathrm{R}^{\otimes m}})$, for some great-enough m. A joint unitary defined on $R^{\otimes m}$ and W approximates $U_{R^{\otimes m}}$ well and uses the system W as a reference frame [Eq. (70)]. This joint unitary conserves every global charge. Because the expectation value of $\mathcal{W}_{\mathrm{R}^{\otimes m}}$ decreases, chemical work is transferred to the battery. □

The NATS is completely passive with respect to $\mathcal{W}_{\mathrm{R}}$ but not necessarily with respect to each charge Q_j. The latter lack of passivity was viewed as problematic in [18]. The lowering of the NATS's $\langle Q_j\rangle$'s creates no problems in our framework, because free operations cannot lower the NATS's $\langle\mathcal{W}\rangle$. The possibility of extracting charge of a desired type Q_j, rather than energy, is investigated also in [29].

For example, let the Q_j's be the components J_j of the spin operator $\mathbf{J}$. Let the z-axis point in the direction of $\boldsymbol{\mu}$, and let $\mu_z > 0$:

$$\sum_{j=1}^{3}\mu_j J_j \equiv \mu_z J_z. \tag{74}$$

The NATS has the form $\rho_{\mathrm{R}} = e^{-\beta(H_{\mathrm{R}}-\mu_z J_{z_{\mathrm{R}}})}/Z$. This ρ_{R} shares an eigenbasis with $J_{z_{\mathrm{R}}}$. Hence the expectation value of the battery's J_x charge vanishes: $\mathsf{Tr}(\rho_{\mathrm{R}}J_{x_{\mathrm{R}}}) = 0$. A free unitary, defined on R and W, can rotate the spin operator that appears in the exponential of ρ_{R}. Under this unitary, the eigenstates of ρ_{R} become eigenstates of $J_{x_{\mathrm{R}}}$. $\mathsf{Tr}(J_x\rho_{\mathrm{R}})$ becomes negative; work appears appears to be extracted "along the J_x-direction" from ρ_{R}. Hence the NATS appears to lack completely passivity. The unitary, however, extracts no chemical work: The decrease in $\mathsf{Tr}(\rho_{\mathrm{R}}J_{x_{\mathrm{R}}})$ is compensated for by an increase in $\mathsf{Tr}(\rho_{\mathrm{R}}J_{z_{\mathrm{R}}})$.

Another example concerns the charges J_i and $\rho_{\mathrm{R}} = e^{-\beta(H_{\mathrm{R}}-\mu_z J_{z_{\mathrm{R}}})}/Z$. No amount of the charge J_z can be extracted from ρ_{R}. But the eigenstates of $-J_z$ are inversely populated: The eigenstate $|z\rangle$ associated with the low eigenvalue $-\frac{\hbar}{2}$ of $-J_z$ has the small population $e^{-\beta\hbar/2}$. The eigenstate $|-z\rangle$ associated with the large eigenvalue $\frac{\hbar}{2}$ of $-J_z$ has the large population $e^{\beta\hbar/2}$. Hence the charge $-J_z$ can be extracted from ρ_{R}. This extractability does not prevent ρ_{R} from being completely passive, according our definition. Only the extraction of $\mathcal{W}$ corresponds to chemical work. The extraction of just one charge does not.

The interconvertibility of types of free energy associated with commuting charges was noted in [17]. Let Q_1 and Q_2 denote commuting charges, and let $\rho_{\mathrm{R}} = e^{-\beta(H_{\mathrm{R}}-\mu_1 Q_{1_{\mathrm{R}}}-\mu_2 Q_{2_{\mathrm{R}}})}$. One can extract Q_1 work at the expense of Q_2 work, by swapping Q_1 and Q_2 (if an allowed unitary implements the swap).

B. Non-Abelian Thermal Operations preserve the Non-Abelian Thermal State.

The NATS, we have shown, is the only completely passive state. It is also the only state preserved by NATO.

Theorem 8. *Consider the resource theory, defined by NATO, associated with a fixed β. Let each free state be specified by $(\rho_{\mathrm{R}}, H_{\mathrm{R}}, Q_{1_{\mathrm{R}}}, \ldots, Q_{c_{\mathrm{R}}})$, wherein $\rho_{\mathrm{R}} := e^{-\beta\,(H_{\mathrm{R}}-\sum_{j=1}^{c}\mu_j Q_{j_{\mathrm{R}}})}/Z$. Suppose that the agent has access to the battery, associated with the payoff function (68). The agent cannot, at a cost of $\langle\mathcal{W}\rangle \leq 0$, transform any number of copies of free states into any other state. In particular, the agent cannot change the state's β or μ_j's.*

Proof. Drawing on Theorem 7, we prove Theorem 8 by contradiction. Imagine that some free operation could transform some number m of copies of $\gamma_{\mathbf{v}} := e^{-\beta\,(H_{\mathrm{R}}-\sum_j \mu_j Q_{j_{\mathrm{R}}})}/Z$ into some other state $\gamma'_{\mathbf{v}}$: $\gamma_{\mathbf{v}}^{\otimes m} \mapsto \gamma'_{\mathbf{v}}$. ($\gamma'_{\mathbf{v}}$ could have a different form from the NATS's. Alternatively, $\gamma'_{\mathbf{v}}$ could have the same form but have different μ_j's or a different β.) $\gamma'_{\mathbf{v}}$ is not completely passive. Work could be extracted from some number n of copies of $\gamma'_{\mathbf{v}}$, by Theorem 7. By converting copies of $\gamma_{\mathbf{v}}$ into copies of $\gamma'_{\mathbf{v}}$, and extracting work from copies of $\gamma'_{\mathbf{v}}$, the agent could extract work from $\gamma_{\mathbf{v}}$ for free. But work cannot be extracted from $\gamma_{\mathbf{v}}$, by Theorem 7. Hence $\gamma_{\mathbf{v}}^{\otimes m}$ must not be convertible into any $\gamma'_{\mathbf{v}} \neq \gamma_{\mathbf{v}}$, for all $m = 1, 2, \ldots$. □

Second laws: Consider any resource theory defined by operations that preserve some state, e.g., states of the form $e^{-\beta\,(H_{\mathrm{R}}-\sum_{j=1}^{c}\mu_j Q_{j_{\mathrm{R}}})}/Z$. Consider any distance measure on states that is contractive under the free operations. Every

state's distance from the preserved state $\rho_{\rm R}$ decreases monotonically under the operations. NATO can be characterized with any distance measure from $\rho_{\rm R}$ that is contractive under completely positive trace-preserving maps. We focus on the Rényi divergences, extending the second laws developed in [26] for the resource theory for heat exchanges.

To avoid excessive subscripting, we alter our notation for the NATS. For any subsystem T, we denote by γ_T the NATS relative to the fixed β, to the fixed μ_j's, and to the Hamiltonian H_T and the charges $Q_{1_T}, \ldots, Q_{c_T}$ associated with T. For example, $\gamma_{\rm SW} := e^{-\beta[(H_{\rm S}+H_{\rm W})+\sum_{j=1}^{c}\mu_j(Q_{j_{\rm S}}+Q_{j_{\rm W}})]}/Z$ denotes the NATS associated with the system-and-battery composite.

We define the generalized free energies

$$F_\alpha(\rho_{\rm S}, \gamma_{\rm S}) := k_{\rm B}T D_\alpha(\rho_{\rm S}\|\gamma_{\rm S}) - k_{\rm B}T\log(Z). \tag{75}$$

The classical Rényi divergences $D_\alpha(\rho_{\rm S}\|\gamma_{\rm S})$ are defined as

$$D_\alpha(\rho_{\rm S}\|\gamma_{\rm S}) := \frac{\operatorname{sgn}(\alpha)}{\alpha-1}\log\left(\sum_k p_k^\alpha q_k^{1-\alpha}\right), \tag{76}$$

wherein p_k and q_k denote the probabilities of the possible outcomes of measurements of the work function $\mathcal{W}$ associated with $\rho_{\rm S}$ and with $\gamma_{\rm S}$. The state $\rho_{\rm S}$ of S is compared with the NATS associated with $H_{\rm S}$ and with the $Q_{j_{\rm S}}$'s.

The F_α's generalize the thermodynamic free energy. To see how, we consider transforming n copies $(\rho_{\rm S})^{\otimes n}$ of a state $\rho_{\rm S}$. Consider the asymptotic limit, similar to the thermodynamic limit, in which $n \to \infty$. Suppose that the agent has some arbitrarily small, nonzero probability ε of failing to achieve the transformation. ε can be incorporated into any F_α via "smoothing" [26]. The smoothed F_α^ε per copy of $\rho_{\rm S}$ approaches F_1 in the asymptotic limit [26]:

$$\lim_{n\to\infty}\frac{1}{n}F_\alpha^\varepsilon\Big((\rho_{\rm S})^{\otimes n}, (\gamma_{\rm S})^{\otimes n}\Big) = F_1(\rho_{\rm S}) \tag{77}$$

$$= \langle H_{\rm S}\rangle_{\rho_{\rm S}} - TS(\rho_{\rm S}) + \sum_{j=1}^{c}\mu_j\langle Q_{j_{\rm S}}\rangle. \tag{78}$$

This expression resembles the definition $F := E - TS + \sum_{j=1}^{c}\mu_j Q_j$ of a thermodynamic free energy F. In terms of these generalized free energies, we formulate second laws.

Proposition 9. *In the presence of a heat bath of inverse temperature β and chemical potentials μ_j, the free energies $F_\alpha(\rho_{\rm S}, \gamma_{\rm S})$ decrease monotonically:*

$$F_\alpha(\rho_{\rm S}, \gamma_{\rm S}) \geq F_\alpha(\rho'_{\rm S}, \gamma_{\rm S}) \;\; \forall\alpha \geq 0, \tag{79}$$

wherein $\rho_{\rm S}$ and $\rho'_{\rm S}$ denote the system's initial and final states. If

$$[\mathcal{W}_{\rm S}, \rho'_{\rm S}] = 0 \quad \text{and}$$
$$F_\alpha(\rho_{\rm S}, \gamma_{\rm S}) \geq F_\alpha(\rho'_{\rm S}, \gamma_{\rm S}) \;\; \forall\alpha \geq 0, \tag{80}$$

some catalytic NATO maps $\rho_{\rm S}$ to $\rho'_{\rm S}$.

The $F_\alpha(\rho_{\rm S}, \gamma_{\rm S})$'s are called "monotones." Under NATO, the functions cannot increase. The transformed state approaches the NATS or retains its distance.

Two remarks about extraneous systems are in order. First, the second laws clearly govern operations during which no work is performed on the system S. But the second laws also govern work performance: Let SW denote the system-and-battery composite. The second laws govern the transformations of SW. During such transformations, work can be transferred from W to S.

Second, the second laws govern transformations that change the system's Hamiltonian. An ancilla facilitates such transformations [15]. Let us model the change, via external control, of an initial Hamiltonian $H_{\rm S}$ into $H'_{\rm S}$. Let $\gamma_{\rm S}$ and ${\gamma_{\rm S}}'$ denote the NATSs relative to $H_{\rm S}$ and to $H'_{\rm S}$. The second laws become

$$F_\alpha(\rho_{\rm S}, \gamma_{\rm S}) \geq F_\alpha(\rho'_{\rm S}, {\gamma_{\rm S}}') \;\; \forall\alpha \geq 0. \tag{81}$$

Extractable work: In terms of the free energies, we can bound the work extractable from a resource state via NATO. Unlike in the previous section, we consider the battery W separately from the system S of interest. We assume that W and S initially occupy a product state. (This assumption is reasonable for the idealised, infinite-dimensional

battery we have been considering. As we will show, the assumption can be dropped when we focus on average work.) Let $\rho_{\rm W}$ and $\rho'_{\rm W}$ denote the battery's initial and final states. For all α,

$$F_\alpha(\rho_{\rm S} \otimes \rho_{\rm W}, \gamma_{\rm SW}) \geq F_\alpha(\rho'_{\rm S} \otimes \rho'_{\rm W}, \gamma_{\rm SW}). \tag{82}$$

Since $F_\alpha(\rho_{\rm S} \otimes \rho_{\rm W}, \gamma_{\rm SW}) = F_\alpha(\rho_{\rm S}, \gamma_{\rm S}) + F_\alpha(\rho_{\rm W}, \gamma_{\rm W})$,

$$F_\alpha(\rho'_{\rm W}, \gamma_{\rm W}) - F_\alpha(\rho_{\rm W}, \gamma_{\rm W}) \leq F_\alpha(\rho_{\rm S}, \gamma_{\rm S}) - F_\alpha(\rho'_{\rm S}, \gamma_{\rm S}). \tag{83}$$

If the battery states $\rho_{\rm W}$ and $\rho'_{\rm W}$ are energy eigenstates, the left-hand side of Ineq. (83) represents the work extractable during one implementation of the protocol. Hence the right-hand side bounds the work extractable during the transition $\rho_{\rm S} \mapsto \rho'_{\rm S}$. This bound is a necessary condition under which work can be extracted.

When $\alpha = 1$, we need not assume that W and S occupy a product state. The reason is that subadditivity implies $F_1(\rho_{\rm SW}, \gamma_{\rm SW}) \leq F_1(\rho_{\rm S}, \gamma_{\rm S}) + F_1(\rho_W, \gamma_{\rm W})$. F_1 is the relevant free energy if only the average work is important.

Quantum second laws: As in [26], additional laws can be derived in terms of quantum Rényi divergences [30–33]. These laws provide extra constraints if $\rho_{\rm S}$ (and/or $\rho'_{\rm S}$) has coherences relative to the $\mathcal{W}_{\rm S}$ eigenbasis. Such coherences would prevent $\rho_{\rm S}$ from commuting with the work function. Such noncommutation is a signature of truly quantum behavior. Two quantum analogues of $F_\alpha(\rho_{\rm S}, \gamma_{\rm S})$ are defined as

$$\tilde{F}_\alpha(\rho_{\rm S}, \gamma_{\rm S}) := k_{\rm B}T \frac{\mathrm{sgn}(\alpha)}{\alpha - 1} \log\left(\mathsf{Tr}\left(\rho_{\rm S}^\alpha (\gamma_{\rm S})^{1-\alpha} \right) \right) - k_{\rm B}T \log(Z) \tag{84}$$

and

$$\hat{F}_\alpha(\rho_{\rm S}, \gamma_{\rm S}) := k_{\rm B}T \frac{1}{\alpha - 1} \log\left(\mathsf{Tr}\left((\gamma_{\rm S})^{\frac{1-\alpha}{2\alpha}} \rho_{\rm S} (\gamma_{\rm S})^{\frac{1-\alpha}{2\alpha}} \right)^\alpha \right) - k_{\rm B}T \log(Z). \tag{85}$$

The additional second laws have the following form.

Proposition 10. *NATO can transform $\rho_{\rm S}$ into $\rho'_{\rm S}$ only if*

$$\hat{F}_\alpha(\rho_{\rm S}, \gamma_{\rm S}) \geq \hat{F}_\alpha(\rho'_{\rm S}, \gamma_{\rm S}) \quad \forall \alpha \geq \frac{1}{2}, \tag{86}$$

$$\hat{F}_\alpha(\gamma_{\rm S}, \rho_{\rm S}) \geq \hat{F}_\alpha(\gamma_{\rm S}, \rho_{\rm S}) \quad \forall \alpha \in \left[\frac{1}{2}, 1\right], \quad \text{and} \tag{87}$$

$$\tilde{F}_\alpha(\rho_{\rm S}, \gamma_{\rm S}) \geq \tilde{F}_\alpha(\rho'_{\rm S}, \gamma_{\rm S}) \quad \forall \alpha \in [0, 2]. \tag{88}$$

These laws govern transitions during which the Hamiltonian changes via an ancilla, as in [15].

SUPPLEMENTARY REFERENCES

[1] Ogata, Y. Approximating macroscopic observables in quantum spin systems with commuting matrices. *Journal of Functional Analysis* **264**, 2005–2033 (2013).

[2] Hiai, F., Ohya, M. & Tsukada, M. Sufficiency, KMS condition and relative entropy in von Neumann algebras. *Pacific J. Math.* **96**, 99–109 (1981).

[3] Hoeffding, W. Probability inequalities for sums of bounded random variables. *Journal of the American Statistical Association* **58**, 13–30 (1963).

[4] Winter, A. *Coding Theorems of Quantum Information Theory*. Ph.D. thesis, Universität Bielefeld (1999).

[5] Hayashi, M. Optimal sequence of quantum measurements in the sense of Stein's lemma in quantum hypothesis testing. *Journal of Physics A Mathematical General* **35**, 10759–10773 (2002).

[6] Aleksandrov, A. & Peller, V. Operator hölder–zygmund functions. *Advances in Mathematics* **224**, 910 – 966 (2010).

[7] Pusz, W. & Woronowicz, S. L. Passive states and KMS states for general quantum systems. *Comm. Math. Phys.* **58**, 273–290 (1978).

[8] Lenard, A. Thermodynamical proof of the Gibbs formula for elementary quantum systems. *J. Stat. Phys.* **19**, 575 (1978).

[9] Horodecki R., Horodecki, P., Horodecki, M. & Horodecki, K. Quantum entanglement. *Rev. Mod. Phys.* **81**, 865–942 (2009).

[10] Bartlett, S. D., Rudolph, T. & Spekkens, R. W. Reference frames, superselection rules, and quantum information. *Reviews of Modern Physics* **79**, 555–609 (2007).

[11] Veitch, V., Hamed Mousavian, S. A., Gottesman, D. & Emerson, J. The resource theory of stabilizer quantum computation. *New Journal of Physics* **16**, 013009 (2014).
[12] Winter, A. & Yang, D. Operational resource theory of coherence. *Phys. Rev. Lett.* **116**, 120404 (2016).
[13] Devetak, I., Harrow, A. W. & Winter, A. A Resource Framework for Quantum Shannon Theory. *IEEE Trans. Inf. Theor.* **54**, 4587–4618 (2008).
[14] Janzing, D., Wocjan, P., Zeier, R., Geiss, R. & Beth, T. Thermodynamic cost of reliability and low temperatures: Tightening landauer's principle and the second law. *Int. J. Theor. Phys.* **39**, 2717–2753 (2000).
[15] Horodecki, M. & Oppenheim, J. Fundamental limitations for quantum and nano thermodynamics. *Nature Communications* **4**, 2059 (2013).
[16] Yunger Halpern, N. & Renes, J. M. Beyond heat baths: Generalized resource theories for small-scale thermodynamics. *Phys. Rev. E* **93**, 022126 (2016).
[17] Yunger Halpern, N. Beyond heat baths II: Framework for generalized thermodynamic resource theories. Preprint at http://arxiv.org/abs/1409.7845 (2014).
[18] Lostaglio, M., Jennings, D. & Rudolph, T. Thermodynamic resource theories, non-commutativity and maximum entropy principles. Preprint at http://arxiv.org/abs/1511.04420 (2015).
[19] Kitaev, A., Mayers, D. & Preskill, J. Superselection rules and quantum protocols. *Phys. Rev. A* **69**, 052326 (2004).
[20] Dahlsten, O. C. O., Renner, R., Rieper, E. & Vedral, V. Inadequacy of von Neumann entropy for characterizing extractable work. *New Journal of Physics* **13**, 053015 (2011).
[21] Del Rio, L., Åberg, J., Renner, R., Dahlsten, O. & Vedral, V. The thermodynamic meaning of negative entropy. *Nature* **474**, 61–63 (2011).
[22] Åberg, J. Truly work-like work extraction via single-shot analysis. *Nature Communications* **4**, 1925 (2013).
[23] Brandão, F. G. S. L., Horodecki, M., Oppenheim, J., Renes, J. M. & Spekkens, R. W. Resource theory of quantum states out of thermal equilibrium. *Physical Review Letters* **111**, 250404 (2013).
[24] Åberg, J. Catalytic coherence. *Phys. Rev. Lett.* **113**, 150402 (2014).
[25] Korzekwa, K., Lostaglio, M., Oppenheim, J. & Jennings, D. The extraction of work from quantum coherence. *New Journal of Physics* **18** (2016).
[26] Brandao, F. G. S. L., Horodecki, M., Ng, N. H. Y., Oppenheim, J. & Wehner, S. The second laws of quantum thermodynamics. *Proc. Natl. Acad. Sci.* **112**, 3275–3279 (2015).
[27] Skrzypczyk, P., Short, A. J. & Popescu, S. Work extraction and thermodynamics for individual quantum systems. *Nature Communications* **5**, 4185 (2014).
[28] Lostaglio, M., Korzekwa, K., Jennings, D. & Rudolph, T. Quantum Coherence, Time-Translation Symmetry, and Thermodynamics. *Physical Review X* **5**, 021001 (2015).
[29] Guryanova, Y., Popescu, S., Short, A. J., Silva, R. & Skrzypczyk, P. Thermodynamics of quantum systems with multiple conserved quantities. Preprint at http://arxiv.org/abs/1512.01190 (2015).
[30] Hiai, F., Mosonyi, M., Petz, D. & Bény, C. Quantum f-divergences and error correction. *Rev. Math. Phys.* **23**, 691–747 (2011).
[31] Müller-Lennert, M., Dupuis, F., Szehr, O., Fehr, S. & Tomamichel, M. On quantum Rényi entropies: A new generalization and some properties. *Journal of Mathematical Physics* **54** (2013).
[32] Wilde, M. M., Winter, A. & Yang, D. Strong converse for the classical capacity of entanglement-breaking and Hadamard channels via a sandwiched Rényi relative entropy. *Communications in Mathematical Physics* **331**, 593–622 (2014).
[33] Jaksic, V., Ogata, Y., Pautrat, Y. & Pillet, C.-A. Entropic fluctuations in quantum statistical mechanics. An introduction. In Fröhlich, J., Manfred, S., Vieri, M., De Roeck, W. & Cugliandolo, L. F. (eds.) *Quantum Theory from Small to Large Scales: Lecture Notes of the Les Houches Summer School*, vol. 95 (Oxford University Press, 2012).
